# Supplementary material for: Tetrahedral DNA loaded siCCR2 restrains M1 macrophage polarization to ameliorate pulmonary fibrosis in chemoradiation-induced murine model
Source: Mol Ther. 2024 Jan 24;32(3):766–82. doi: 10.1016/j.ymthe.2024.01.022 (PMC10928155; doi:10.1016/j.ymthe.2024.01.022)
Supplement: Document S1. Figures S1–S7 and Tables S1–S3 [file mmc1.pdf]

YMTHE, Volume *32*

Supplemental Information

Tetrahedral DNA loaded siCCR2 restrains M1 macrophage polarization to ameliorate pulmonary fibrosis in chemoradiation-induced murine model

Chen Li, Xiaorong Feng, Songhang Li, Xing He, Zeli Luo, Xia Cheng, Jie Yao, Jie Xiao, Xiaofei Wang, Dingke Wen, Duanya Liu, Yanfei Li, Hong Zhou, Lu Ma, Tongyu Lin, Xiaoxiao Cai, Yunfeng Lin, Lu Guo, and Mu Yang

Supplemental Material

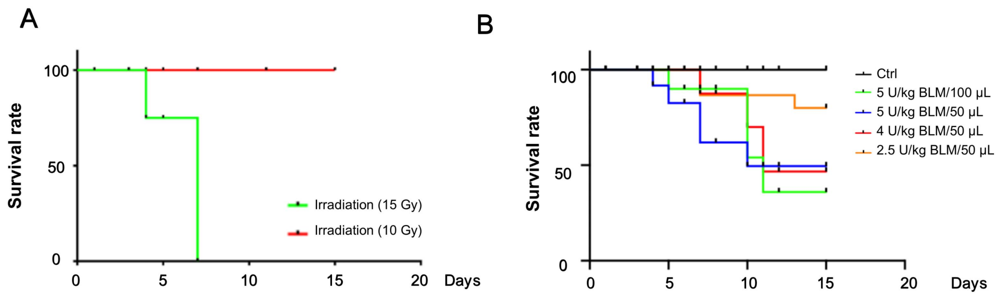

Figure S1. Does selection of IR and BLM. (A and B) Survival analyses after IR (A) and BLM (B) treatment in different dose.

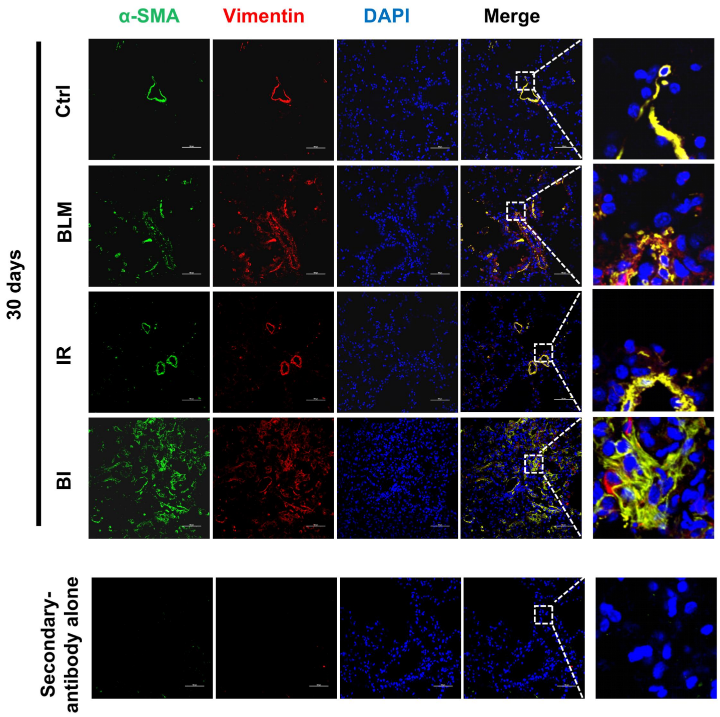

Figure S2. Activated myofibroblasts in pulmonary tissues on day 30 post-induction. Representative immunofluorescence images of α-SMA^{+}(green)Vimentin^{+}(red) myofibroblasts in pulmonary parenchyma of murine models; sections were co-stained by Vimentin (red) and α-SMA (green) antibodies, DAPI as blue nuclear counterstain, bar = 100 μm.

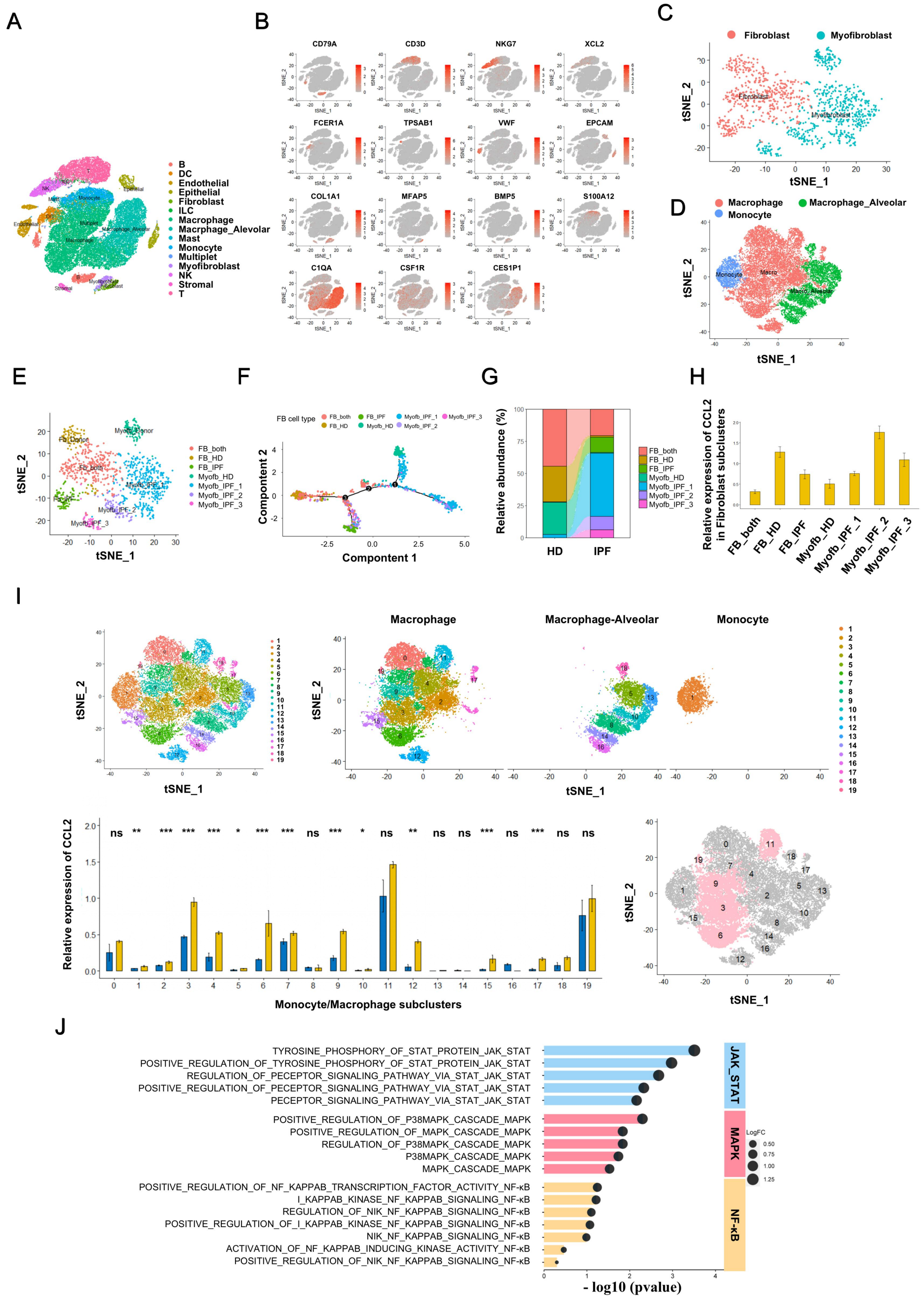

Figure S3. scRNAseq analysis of cell clusters in pulmonary parenchyma of IPF patients. (A) T-distributed stochastic neighbour embedding (t-STE) plot to visualize cell-type clusters based on unsupervised clustering analysis of scRNAseq. (B) Expression profile of selected genes in cell clusters of pulmonary parenchyma. (C)

Clusters of myofibroblast and fibroblast by individual annotation from scRNAseq data. (D) Clusters of monocytes and macrophages by individual annotation from scRNAseq data. (E) Sub-clusters of fibrosis/myofibrosis by individual annotation from scRNAseq data. (F) Pseudotime trajectory of fibrosis/myofibrosis sub-clusters analyzed by Monocle. (G) Relative abundance of fibrosis/myofibrosis sub-clusters. (H) CCL2 expression in selected sub-clusters of fibrosis/myofibrosis. (I) Sub-clusters of monocyte/macrophage by individual annotation from scRNAseq data, and CCL2 expression in selected sub-clusters. (J) KEGG analysis of CCL2/CCR2 axis in macrophage. Data were analyzed using student's *t*-test, $^{*}P < 0.05$, $^{**}P < 0.01$, and $^{***}P < 0.001$ versus positive control.

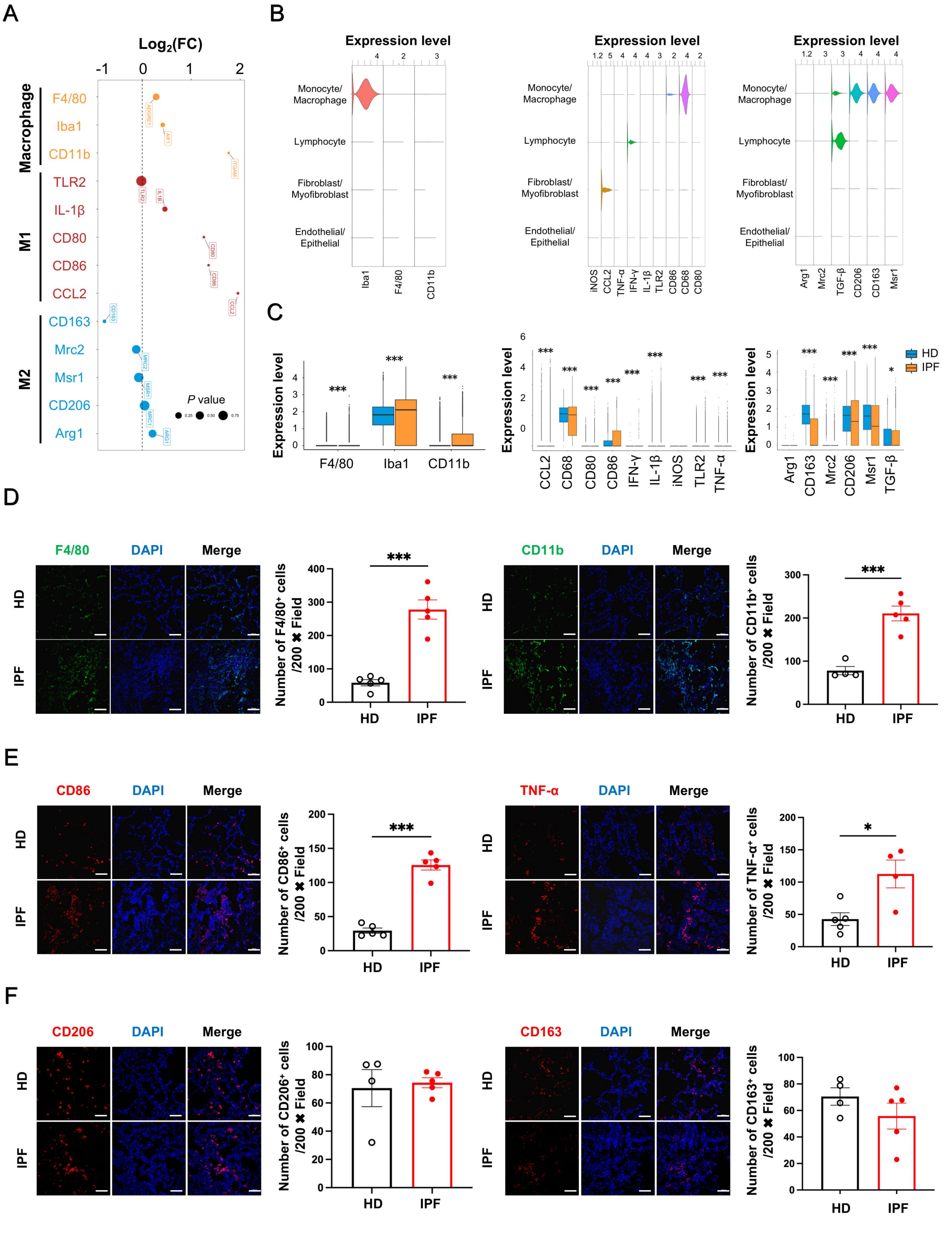

Figure S4. Identification of macrophage in pulmonary parenchymal of IPF patients. (A) Pesudo-bulk differential expression analysis of multiple macrophage parameters in lung between healthy donors and IPF patients (log_2(fold change(FC)) (GSE122960). (B) Multiple macrophage parameter expression levels in different cell clusters of lung. (C) Differential expression of multiple macrophage parameter in the monocyte/macrophage cluster of lung between healthy donors and IPF patients. (D)

Representative immunofluorescence images and quantification analysis F4/80$^+$ and CD11b$^+$ macrophage in lung of healthy donors and IPF patients. (E and F) Representative immunofluorescence images and quantification analysis of CD86$^+$ and TNFα$^+$ M1 macrophage (E) and CD206$^+$ and CD163$^+$(red) M2 macrophage (F) in lung sections of IPF patients and healthy donors. Bar=100 μm, data (*n*=4-5) were expressed as mean±SEM and analyzed using student's *t*-test, $^{*}P < 0.05$ and $^{***}P < 0.001$ between two groups.

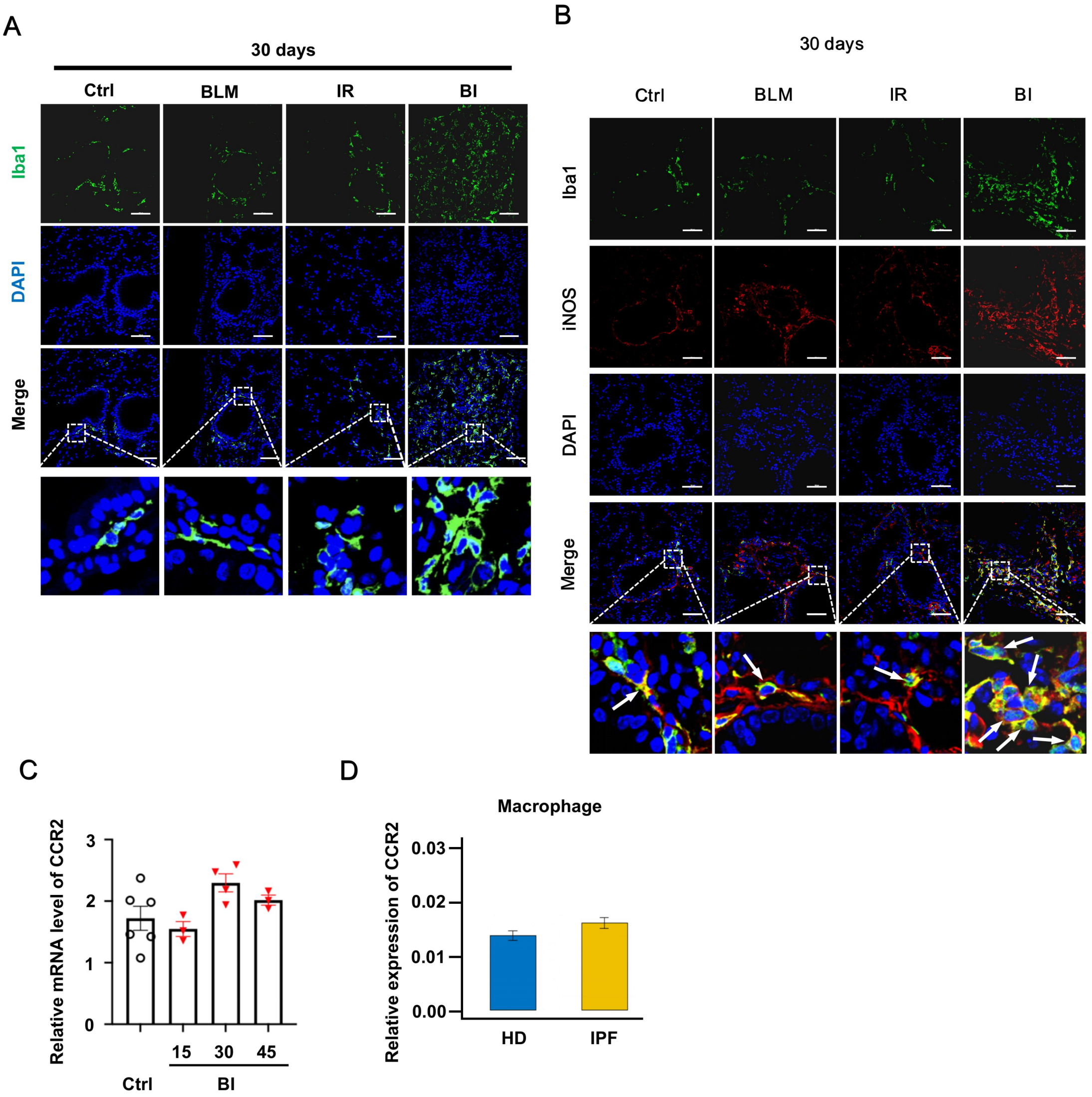

Figure S5. Increased M1 macrophage in pulmonary tissue of murine models on day 30 after induction. (A and B) Representative immunofluorescence images of Iba1^{+}(green) labeled macrophage (A) and Iba1^{+}(green)iNOS^{+}(red) labeled M1 macrophage (B) in pulmonary tissues. (C) The mRNA expression levels of CCR2 in pulmonary tissues from BI group at day 15, 30, and 45 post-induction. (D) The mRNA expression levels of CCR2 in macrophage of pulmonary tissues from HD and IPF patients. Bar=100 μm, data were expressed as mean±SEM and analyzed using one-way ANOVA with Tukey's multiple comparisons test.

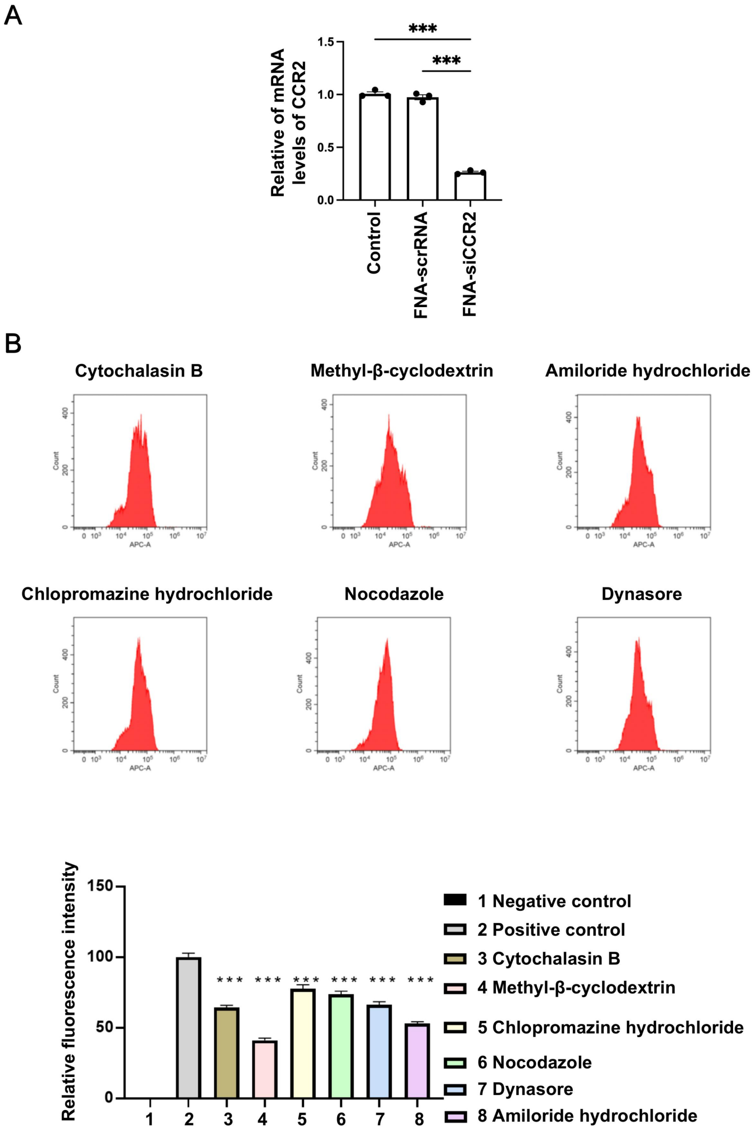

Figure S6. (A) RT-PCR reveal the inhibitory efficacy of FNA-siCCR2 on the CCR2 gene expression in macrophages. (B) Analysis of the amount of FNA-siCCR2-Cy5 endocytosed after adding various inhibitors, n=3 for each group. Data were expressed as mean±SEM and analyzed using student's t-test, $^{***}P < 0.001$ between two groups or versus positive control.

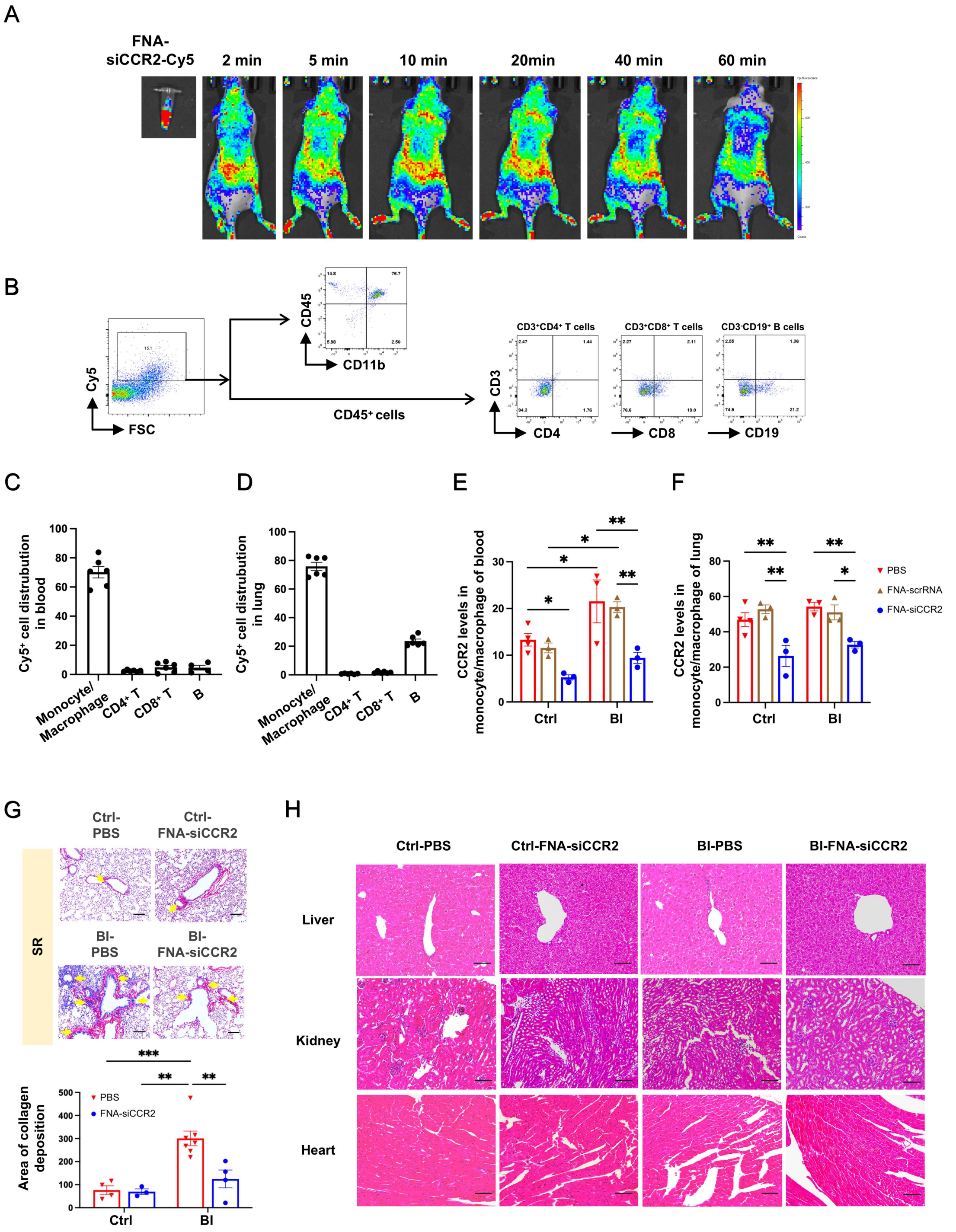

Figure S7. FNA-siCCR2 prevent pulmonary fibrosis in IPF murine model. (A) Continuously observation of FNA-siCCR2-Cy5 signals in mice after i.v. injection by in *vivo* imaging system. (B) Representative flow cytometry plots showing gating strategy for $CD45^{+}CD11b^{+}$ monocyte/macrophage, $CD3^{+}CD4^{+}$ T cells, $CD3^{+}CD8^{+}$ T cells, and $CD3^{-}CD19^{+}$ B cells in the PBMCs. (C and D) The cell distribution of $Cy5^{+}$ signals in blood and lung tissues after i.v. injection of FNA-siCCR2-Cy5. (E and F) Flow cytometry reveal the inhibitory efficacy of FNA-siCCR2 on the CCR2 gene

expression in monocyte/macrophages of blood and lung tissues. (G) The representative images of SR staining and statistically analysis of collagen deposition in lung tissues from murine IPF models after FNA-siCCR2 treated, bar=200 μm, *n*=3-7 for each group. (H) The representative images of HE staining of liver, kidney, and heart tissues from murine IPF model after FNA-siCCR2 treatment, bar=200 μm. Data were expressed as mean±SEM and analyzed using two-way ANOVA with Tukey's multiple comparisons test, $^{*}P < 0.05$ and $^{**}P < 0.01$ between two groups.

Table S1 Clinical details of idiopathic pulmonary fibrosis patients.

Patient NO.	Diagnosis	Gender	Age (y)	Duration (y)	BMI	Smoker	Oxygenation index (mmHg)	6 min walk distance (m)
P1	IPF	Male	59	6	27.38	No	133.33	NA
P2	IPF	Male	57	0.8	16.53	Yes	165.85	120
P3	IPF	Female	65	2	23.73	No	184.44	135
P4	IPF	Male	69	2	24.91	Yes	165.85	NA
P5	IPF	Male	62	1.6	21.22	Yes	248.65	NA

NA: Not available

Table S2 Clinical details of healthy donors.

Healthy donor NO.	Gender	Age (y)	Oxygenation index (mmHg)	6 min walk distance (m)
HD1	Male	58	385.71	480
HD2	Male	72	360.71	423
HD3	Female	63	382.14	400
HD4	Male	64	375	468
HD5	Male	66	417.86	416

Table S3 Clinical details of donors for scRNAseq from GSE136831.

	Samples NO.	Diagnosis	Gender	Age
1	GSM4058905	Control	M	50
2	GSM4058907	Control	M	56
3	GSM4058911	Control	M	65
4	GSM4058912	Control	M	64
5	GSM4058914	Control	F	66
6	GSM4058915	Control	F	66
7	GSM4058916	Control	M	61
8	GSM4058917	Control	F	62
9	GSM4058918	Control	M	35
10	GSM4058921	Control	F	67
11	GSM4058923	Control	F	66
12	GSM4058926	Control	M	54
13	GSM4058948	IPF	M	56
14	GSM4058950	IPF	F	66
15	GSM4058952	IPF	F	67
16	GSM4058954	IPF	F	65
17	GSM4058957	IPF	F	67
18	GSM4058960	IPF	M	56
19	GSM4058961	IPF	M	61
20	GSM4058963	IPF	M	65
21	GSM4058969	IPF	M	64
22	GSM4058972	IPF	F	66
23	GSM4058974	IPF	M	54
