# Supplementary material for: Tetrahedral DNA loaded siCCR2 restrains M1 macrophage polarization to ameliorate pulmonary fibrosis in chemoradiation-induced murine model
Source: Mol Ther. 2024 Jan 24;32(3):766–82. doi: 10.1016/j.ymthe.2024.01.022 (PMC10928155; doi:10.1016/j.ymthe.2024.01.022)
Supplement: Document S2. Article plus supplemental information [file mmc2.pdf]

Molecular Therapy
Original Article

Tetrahedral DNA loaded siCCR2 restrains M1 macrophage polarization to ameliorate pulmonary fibrosis in chemoradiation-induced murine model

Chen Li,[1,11] Xiaorong Feng,[1,11] Songhang Li,[2,11] Xing He,[3] Zeli Luo,[4] Xia Cheng,[1] Jie Yao,[1] Jie Xiao,[1] Xiaofei Wang,[5] Dingke Wen,[6] Duanya Liu,[1] Yanfei Li,[7] Hong Zhou,[8] Lu Ma,[6] Tongyu Lin,[1] Xiaoxiao Cai,[2,9] Yunfeng Lin,[2,9] Lu Guo,[10] and Mu Yang[1]

[1]Centre for Translational Research in Cancer, Sichuan Cancer Hospital & Institute, School of Medicine, University of Electronic Science and Technology of China, Chengdu 610042, China; [2]State Key Laboratory of Oral Diseases, National Clinical Research Center for Oral Diseases, West China Hospital of Stomatology, Sichuan University, Chengdu 610041, China; [3]School of Clinical Medicine, Chengdu Medical College, Chengdu 610500, China; [4]Department of Pulmonary and Critical Care Medicine, Wenjiang Hospital of Sichuan Provincial People's, Chengdu 611138, China; [5]Department of Neurology, West China Hospital, Sichuan University, Chengdu 610041, China; [6]Department of Neurosurgery, West China Hospital, Sichuan University, Chengdu 610041, China; [7]School of Basic Medical Sciences, Chengdu University of Traditional Chinese Medicine, Chengdu 610075, China; [8]School of Life Science and Technology, University of Electronic Science and Technology of China, Chengdu 610056, China; [9]College of Biomedical Engineering, Sichuan University, Chengdu 610041, China; [10]Department of Pulmonary and Critical Care Medicine, Sichuan Provincial People's Hospital, School of Medicine, University of Electronic Science and Technology of China, Chengdu 610072, China

Idiopathic pulmonary fibrosis (IPF) is a chronic lethal disease in the absence of demonstrated efficacy for preventing progression. Although macrophage-mediated alveolitis is determined to participate in myofibrotic transition during disease development, the paradigm of continuous macrophage polarization is still under-explored due to lack of proper animal models. Here, by integrating 2.5 U/kg intratracheal Bleomycin administration and 10 Gy thorax irradiation at day 7, we generated a murine model with continuous alveolitis-mediated fibrosis, which mimics most of the clinical features of our involved IPF patients. In combination with data from scRNA-seq of patients and a murine IPF model, a decisive role of CCL2/CCR2 axis in driving M1 macrophage polarization was revealed, and M1 macrophage was further confirmed to boost alveolitis in leading myofibroblast activation. Multiple sticky-end tetrahedral framework nucleic acids conjunct with quadruple ccr2-siRNA (FNA-siCCR2) was synthesized in targeting M1 macrophages. FNA-siCCR2 successfully blocked macrophage accumulation in pulmonary parenchyma of the IPF murine model, thus preventing myofibroblast activation and leading to the disease remitting. Overall, our studies lay the groundwork to develop a novel IPF murine model, reveal M1 macrophages as potential therapeutic targets, and establish new treatment strategy by using FNA-siCCR2, which are highly relevant to clinical scenarios and translational research in the field of IPF.

INTRODUCTION

As one of the most common respiratory diseases with devastating prognosis, idiopathic pulmonary fibrosis (IPF) mainly leads to alveolar dysfunction and lung failure.[1,2] Up to 50% of IPF patients die within 3 years in the absence of demonstrated efficacy in preventing disease progression.[3,4] Etiologically, environmental factors, viral infection, medication, and radiation pneumonia are believed to make patients more predisposed to IPF.[5–8] Those exogenous stimuli mainly induce alveolitis, which has been highlighted in recent studies for its contributions in triggering myofibrotic transition at early stages.[9–11] Thus, other than alveolar epithelial type II cell (AT2) imbalance, dynamic changes of immune cells involved in alveolitis were thought to be responsible for IPF development as well.[10–12] Activation and polarization of macrophages are involved in inflammation, which play a pivotal role in the pathogenesis of pulmonary fibrosis disease.[13–16] Classical/M1 macrophage exhibit a pro-inflammatory phenotype, which is characterized by high levels of inducible NO synthase (iNOS), pro-inflammatory cytokines TNF-α and IL6, and chemokines CCL2.[17–20] The factors released from these cells trigger local inflammatory responses as well as activation of fibrosis.[17–20] Alternative/M2 macrophages are involved in an anti-inflammatory phenotype and repairing inflammation-associated injury, which is characterized by high levels of Arginase-1 and

Received 7 September 2023; accepted 18 January 2024;
https://doi.org/10.1016/j.ymthe.2024.01.022.

[11]These authors contributed equally

Correspondence: Yunfeng Lin, State Key Laboratory of Oral Diseases, National Clinical Research Center for Oral Diseases, West China Hospital of Stomatology, Sichuan University, Chengdu 610041, China.
E-mail: yufenglin@scu.edu.cn
Correspondence: Lu Guo, Department of Pulmonary and Critical Care Medicine, Sichuan Provincial People's Hospital, School of Medicine, University of Electronic Science and Technology of China, Chengdu 610072, China.
E-mail: guoluhx@med.uestc.edu.cn
Correspondence: Mu Yang, Centre for Translational Research in Cancer, Sichuan Cancer Hospital & Institute, School of Medicine, University of Electronic Science and Technology of China, Chengdu 610042, China.
E-mail: mu.yang@uestc.edu.cn

 © 2024 The Author(s).
This is an open access article under the CC BY license (http://creativecommons.org/licenses/by/4.0/).

anti-inflammatory mediator IL-10, TGF-β, CD36, and Nrf2.[21–23] Notably, both the infiltrating and resident macrophage state within pulmonary mesenchyma that express key markers of M1 phenotype were demonstrated to connect alveolitis and mesenchymal transition, thus enabling expansion of the fibrotic site in lung.[13–20] Although the aforementioned clinical observations provide good insight into the relationship between pulmonary alveolar fibroblasts and/or activating myofibroblasts with macrophage-induced inflammatory cascades in IPF patients. But given the lack of a proper innate immunity-triggered animal model with progressive IPF, inadequate evidence is obtained about continuous inspections during disease progression, such as the mechanism in driving M1 macrophage polarization in lung and continuous alveolitis-mediated myofibroblast transition from fibroblasts, which are considered as major obstacles to finding a new therapeutic target, as well as drug development for disease prevention.

Due to the critical clinical needs for identification of the conversion from alveolar fibroblast to myofibroblast under constitutive alveolitis, IPF murine models are thought to construct for longitudinally monitoring macrophage polarization manipulated fibrosis.[13–15,24] Previous reports indicated that irradiation (IR)-mediated pulmonary fibrosis rodent model could partially recapitulate the histopathological features of IPF, including progressive pulmonary fibrosis with irreversible characteristics, but less immune cell infiltration was found at disease onset except for acute inflammation mediated by IR.[25–27] On the other hand, Bleomycin (BLM) as the most commonly used chemical to trigger IPF murine model, its ability in leading lung fibrosis mainly lies on dose-dependent toxicities, which mostly bring self-limited inflammation via DAMP activates myloid cells.[28,29] Based on the progressive and nonreversible features of IPF that was observed in clinical practices, BLM triggered IPF murine model may share a different mechanism with chronic alveolitis starting from the early stage of IPF.[2,3,25,28,29] The latest report from *cdc42* knockout mice also implied a genetic possibility of IPF onset in blocking regeneration of alveolar by activating AT2, which mimics most pathological features of IPF patients at the late stage.[30] Nevertheless, by lack of considerable *in situ* inflammation and prominent exogenous stimuli, preliminary information for the extended dynamics of immune cells in driving fibrotic cascades was elucidated.[30] In considering the indistinct boundary between chronic inflammation and disease progression of IPF patients, obvious immune activation and inexorable fibrosis with prominent symptoms should be considered as the features of IPF murine models.[2,12,25]

The multiple sticky-end tetrahedral framework nucleic acids (FNAs) are synthesized by four single DNA strands through self-assembly based on the Watson-Crick base pairing rule.[31] This tetrahedral DNA has been used extensively in drug delivery due to its excellent biological characteristics, such as simple synthesis, stability, low toxicity, tissue permeability, and editability.[32] In particular, FNAs possess several modification sites that can load therapeutic oligonucleotides (siRNA or miRNA), which could be endocytosed by myeloid cells in a caveolin-dependent manner to achieve the effect of gene expression regulation at the post-transcriptional level.[31,33] To date, except for an inhaled ribosomal protein-based mRNA nanoformulation used to deliver the mRNA of MMP13 for the treatment of BLM-induced pulmonary fibrosis, the therapeutic efficacy of FNA-delivered oligonucleotides for IPF remains unknown.[34]

The aim of our present study was to construct a new IPF murine model that mimics the irreversible pathological changes and clinical features of IPF patients, thus tackled both self-limiting symptoms and alveolitis-free fibrosis in current IPF murine models. Following behavior and pathological evaluations exhibited progressive respiratory failure and pulmonary fibrosis of this chemoradiation-induced mice with constitutive inflammation that start from early stages. Consistent with our comprehensive single-cell RNA sequencing (scRNA-seq) data from involved IPF patients, increased macrophages residing at lesion sites were found to leverage the development of IPF in this murine model as well. Furthermore, a potential mechanism of IPF, as well as the novel therapeutic target were revealed in relation to continuous activation of myofibroblasts. Observations revealed a decisive role of the CCL2/CCR2 axis in driving M1 macrophage polarization, and this type of macrophages was confirmed to boost alveolitis in leading myofibroblast activation. By employing FNA thus specifically delivering quadruple *ccr2*-siRNA into monocytes/macrophages (FNA-siCCR2), CCL2 failed to recruit monocytes/macrophages, as well as M1 polarization. Alveolitis-derived severe pulmonary fibrosis was determined to be successfully prevented without M1 macrophage participation in an IPF murine model under FNA-siCCR2 administration.

RESULTS

Establishment and evaluation of IPF murine model

BLM in combined with IR was employed to generate pulmonary inflammation-mediated fibrosis in C57BL6 mice (Figures 1A, and S1). As both excessive doses of IR or BLM would mediate fatal symptoms before fibrosis derivation, respectively (Figure S1), 2.5 U/kg BLM with 10 Gy whole thorax IR at day 7 were adopted to establish our current setting (Figure 1A). Without lethal exposure of irradiation, the survival rate of BLM + IR (BI) group (68%) was not significantly different from the BLM group (72%) until 45 days post-induction (Figure 1B). Except a trend of respiratory abilities decreasing on day 30 and 45 after derivation, respectively, no obvious body weight and pulmonary function changes were monitored in BLM or IR-induced mice (Figures 1C and 1D). Nevertheless, the BI group showed consistent body weight loss starting from day 26, as well as continuous reduction of the distance on the treadmill running to exhaustion, which indicated a pulmonary dysfunction (Figures 1C and 1D).

Previous studies revealed that massive collage deposition in the extracellular matrix is the primary characteristic of IPF in Modified Ashcroft scores (MASs).[35–37] Therefore, massive collagen deposition and elevated MAS implied the damage of alveolar structure thus induced pulmonary dysfunction. Pathologically, BI-treated mice exhibited progressive aggravation of alveolar structure damage, mononuclear cells increasing and collagen deposition in lung parenchyma

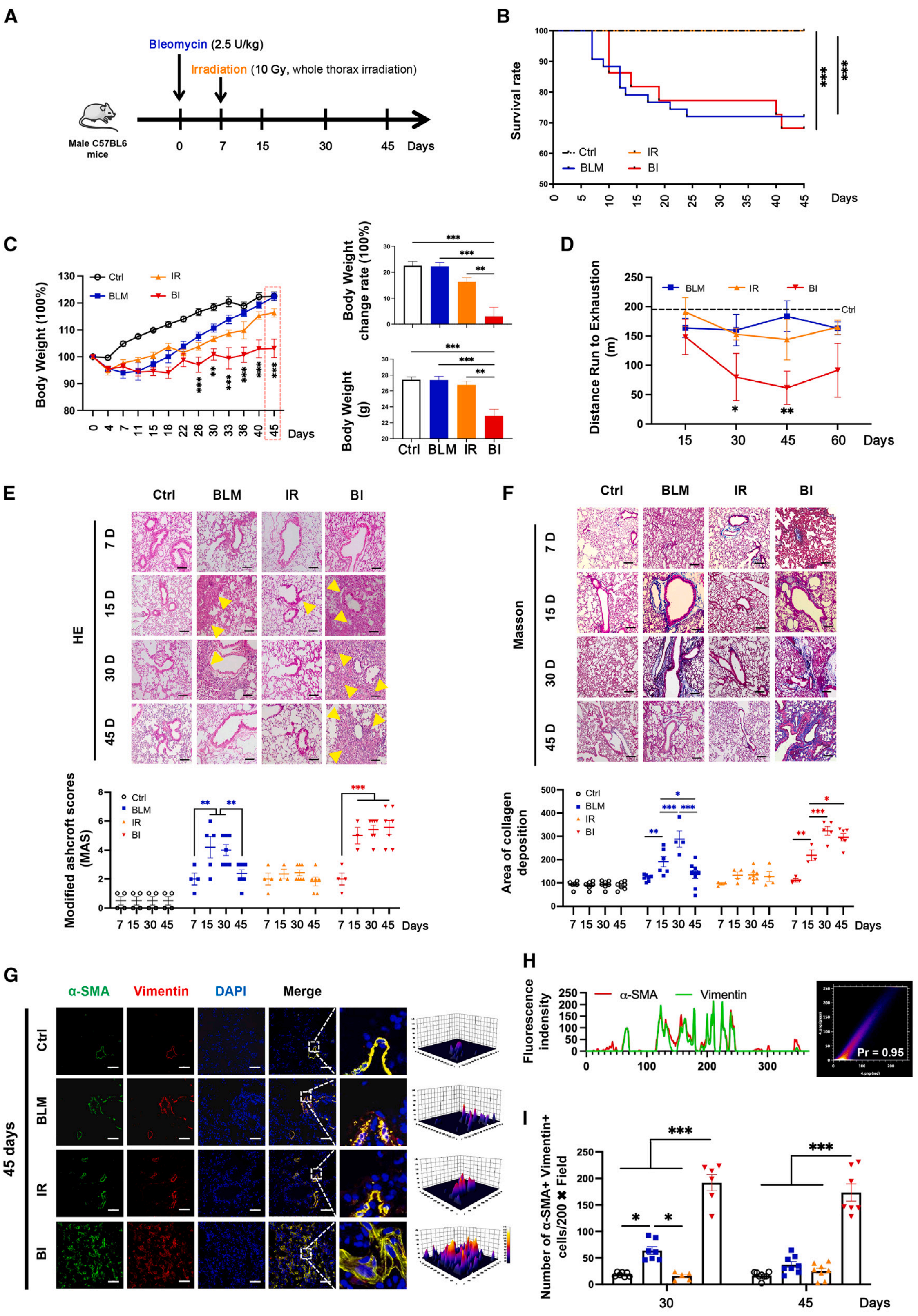

(legend on next page)

starting from day 15 (Figures 1E and 1F). Although less severe pathological damage of lung was observed in BLM- or IR-mediated mice, the lesion site appeared relatively short duration with recovery features after induction (Figures 1E and 1F). Following, as the typical evaluation methods of IPF-mediated pulmonary damage, MASs and collagen deposition area were analyzed among all treated groups from day 7–45 post-induction (Figures 1E and 1F). BI induction brought progressively increasing MAS and collagen deposition at the lesion site until day 45 (Figures 1E and 1F). In parallel, BLM or IR inductions led to moderate elevating of MAS with inconsistent features, whereas collagen deposition was only found in the BLM group at day 15 to day 30 after induction (Figures 1E and 1F). Continuous collagen deposition and elevated MAS damaged the structure and gas exchange function of the alveoli, and the pulmonary dysfunction presents hypokinesia and steady body weight loss in the BI-mediated IPF murine model, but not in the day 45 of BLM-induced murine model. Accordingly, those pathological damages of pulmonary parenchyma might therefore be responsible for the aforementioned alternative behavior changes among BLM, IR, and BI-induced murine model. Lung fibrosis is the ultimate result of excessive collagen deposition, resulting from a series of activation and proliferation of myofibroblasts. Myofibroblast transition is considered as the key feature for the early stages of IPF, we thus explored the accumulation of activating myofibroblast. As specific markers for activated alveolar myofibroblast, the colocalization of α-SMA and Vimentin could particularly represent progressive fibrosis.[38] On day 30 and 45 post-induction, BI group exhibited progressive alveolar myofibroblast activation (Figures 1G–1I and S2). However, these accumulations of α-SMA$^+$Vimentin$^+$ alveolar myofibroblasts were only monitored in the BLM group at day 30, whereas no obvious activation of myofibroblasts was detected in the IR group (Figures 1G–1I and S2).

Taken together, our chemoradiation-induced murine model appeared progressive and irreversible respiratory failure in behavior, and pathological changes including progressive alveolar damage, mononuclear cell accumulation, collagen deposition, as well as activated alveolar myofibroblast increasing that co-localized with fibroblast foci.

Comparable fibrotic characteristics in pulmonary parenchyma of BI-induced mice with IPF patients

To further elucidate the similarity between our murine model and human patients with this ultimately fatal disease, lung tissues were collected from five IPF patients that were prepared to receive lung transplantation (LTx) (Table S1). Five paired healthy donors were also acquired at the same time as well (Table S2). Indeed, in the absence of efficient anti-fibrotic drugs to prevent IPF progression, LTx seems to be the last choice of patients at late stages.[3] Consistent with the typical imaging features of IPF, all patients had suffered severer pulmonary dysfunction, including refractory hypoxemia and dyskinesia due to decrease of both oxygenation index and walking distance (Figures 2A–2C; Tables S1 and S2). Simultaneously, in comparing with lung tissues of healthy donors, IPF patients exhibited significantly higher MAS and collagen deposition in pulmonary parenchyma with alveolar structure damage, mononuclear cell infiltration, and fibroblast foci formation (Figures 2D–2F). Despite of the different methods for clinical evaluation and complications from individual patients in disease severity, currently involved patients had remarkable symptoms and pathological characteristics of IPF (Figures 2D–2F). Following, immunohistofluorescence results demonstrated that massive accumulation of α-SMA$^+$ Vimentin$^+$ myofibroblast in pulmonary parenchyma of IPF patients, and very few of them were found at the comparable locations in lung from healthy donors (Figures 2G–2I). On account of observations from our murine model, α-SMA$^+$Vimentin$^+$ alveolar myofibroblasts also significantly expanded in the lung after BI induction (Figures 1G–1I). Collectively, based on clinical symptoms and pathological changes of IPF patients, similarities were found in our chemoradiation-induced murine model (Figure 1), such as respiratory dysfunction, collagen deposition, and colocalization of activated alveolar myofibroblast with fibroblast foci in pulmonary parenchyma.

Immune landscapes of patients with IPF

Despite α-SMA$^+$ Vimentin$^+$ myofibroblasts increasing, intensive DAPI signals were also exclusively monitored in the pulmonary parenchyma of both IPF patients and the murine model (Figures 1G, and 2G). In considering the latest view from individuals hospitalized with IPF demonstrated that a large number of immune cells are detected in bronchoalveolar lavage fluids (BALF) and lesion sites thus effect on myofibroblast transition,[38–41] scRNA-seq was performed to differ the heterogeneity among immune cells from lung parenchyma of patients with IPF (Figures 3A–3F). With IPF patients and age- and sex-matched healthy donors (HDs) from the Gene Expression Omnibus (GEO) database (Table S3), unsupervised method was applied to partition single-cell transcriptomes into 15 clusters, including parenchymal cells and immune cells with both innate

Figure 1. Behavior changes and pathological features of BLM, IR, and BI-induced murine models

(A) Schematic diagram of the establishment of IPF in mice. (B) Survival curve of mice, Ctrl: n = 28; BLM: n = 43; IR: n = 28; BI: n = 44. (C) Continuous observation of body weight changes of murine models from days 0–45 after induction, Ctrl: n = 22; BLM: n = 19; IR: n = 17; BI: n = 30. (D) The evaluation of maximal endurance running capacity, n = 3–6 for each group of days 15–60. (E) The representative images of H&E staining and statistical analysis of MAS in lung tissues from control group and murine models, bar, 100 μm, n = 3–8 for each group of days 7–45. (F) The representative images of Masson staining and statistical analysis of collagen deposition in lung tissues from control group and murine models; bar, 200 μm, n = 3–9 for each group of days 7–45. (G) Representative immunofluorescence images of α-SMA$^+$ (green) Vimentin$^+$ (red) myofibroblasts in pulmonary parenchymal on day 45 after induction; sections were co-stained by Vimentin(green) and α-SMA(red) antibodies, DAPI as blue nuclear counterstain; bar, 100 μm. (H) Colocalization analysis of α-SMA$^+$ and Vimentin$^+$ in sections (left: the overlap of fluorescence intensity peaks; right: the Pearson coefficient). (I) The quantification analysis of α-SMA$^+$Vimentin$^+$ double-labeled myofibroblasts in lung on days 30 and 45 after induction, respectively, n = 5–8 for each group of days 30 and 45. Data were expressed as mean ± SEM and analyzed using one-way ANOVA with Tukey's multiple comparisons test, *$p < 0.05$, **$p < 0.01$, and ***$p < 0.001$ between two groups.

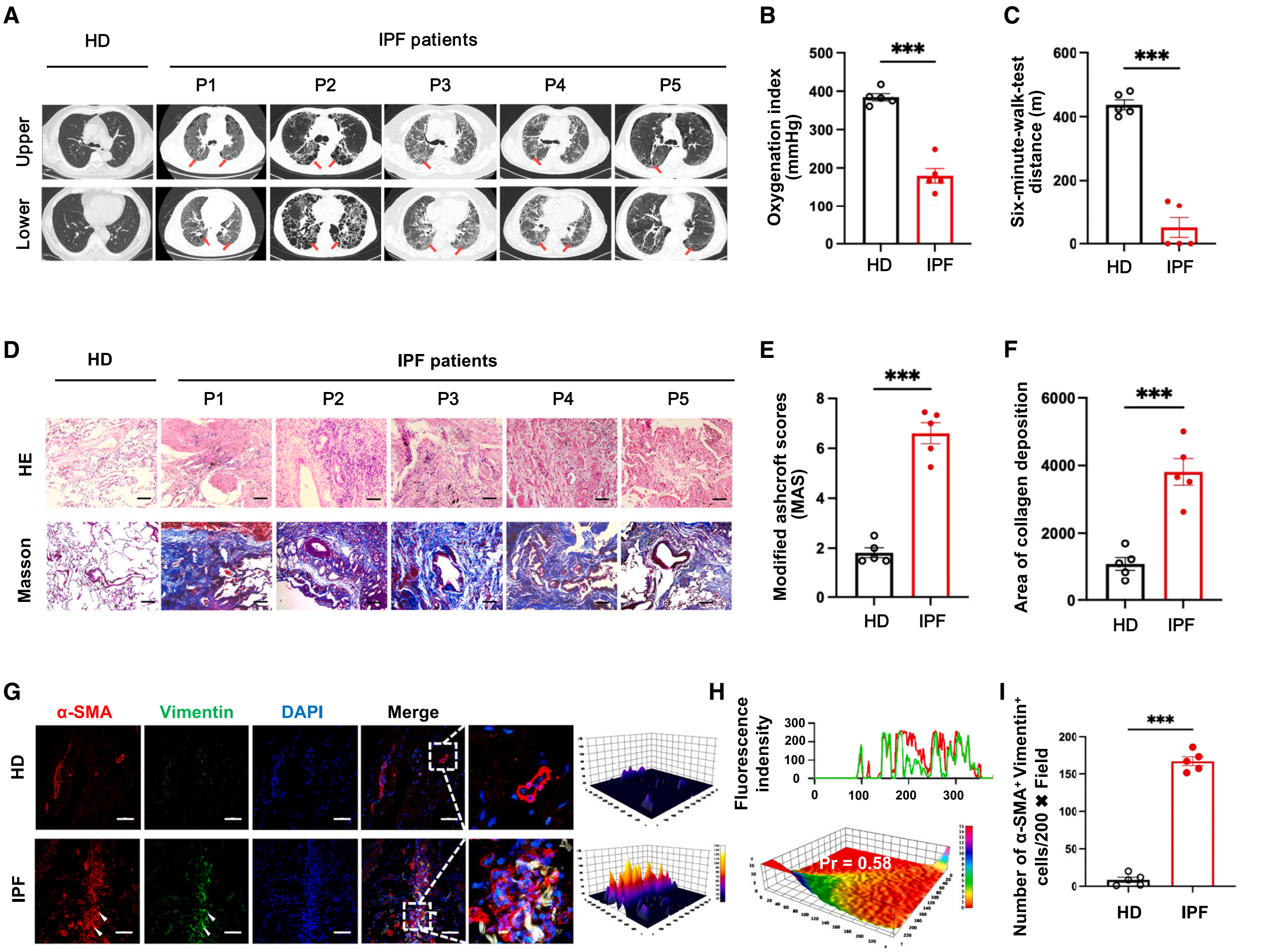

Figure 2. Clinical and pathological features of patients with IPF

(A) The representative images of lung high-resolution computed tomography (HRCT), the red arrows showing typical image features of IPF; P, IPF patient; HD, healthy donor. (B and C) Statistical analysis of oxygenation index (B) and walking distance for 6 min (C) of IPF patients and healthy donors. (D) The representative results of H&E- and Masson-stained lung tissues of HDs and IPF patients; bar, 200 μm. (E and F) The quantification analysis of MAS and collagen deposition of lung tissues. (G) Representative immunofluorescence staining results of α-SMA$^+$Vimentin$^+$ myofibroblasts (white arrowhead) in lung tissues; sections were co-stained by Vimentin (green) and SMA (red) antibodies, DAPI as blue nuclear counterstain; bar, 100 μm. (H) Colocalization analysis of α-SMA$^+$ and Vimentin$^+$ in lung sections (upper: the overlap of fluorescence intensity peaks; lower: 3D heatmap of the Pearson coefficient). (I) The statistical analysis of α-SMA$^+$Vimentin$^+$ cells in the lung section of IPF patients and HDs. Data (HDs: $n = 5$, IPF patients: $n = 5$) were expressed as mean ± SEM and analyzed using Student's t test, $^*p < 0.05$, $^{**}p < 0.01$, and $^{***}p < 0.001$ between two groups.

and adaptive functions (Figures 3A–3C, S3A, and S3B). According to the relative abundance, the most remarkable increasing clusters were myofibroblast from parenchymal subsets of IPF patients, as well as macrophages, which belong to innate immunity (Figure 3C). In particular, myofibroblasts were predominant in the lung of IPF patients, whereas fibrotic cells in lung tissues from HDs was mainly composed of fibroblasts (Figures 3D and S3C). Elevation of macrophage levels was accompanied with slightly alveolar macrophages and monocytes decreasing in IPF patients (Figures 3E and S3D). As a well-known chemotaxis signaling, CCL2/CCR2 is considered to recruit macrophages in triggering inflammation and mediates various inflammatory disease.[42,43] According to our scRNA-seq analysis for IPF patients, enhanced expression of CCL2 was found in myofibroblasts and macrophages from IPF patients (Figures 3F and S3E–S3I). Previous reports in IPF patients considered that alveolar myofibroblasts with α-SMA and Vimentin expression represent the early stages of IPF development and is likely from the transmitting of alveolar epithelium via imbalance of activating AT2.38. However, all patients involved in current studies were diagnosed at the late stages of IPF, whereas epithelial-mesenchymal transition (EMT) should be the most predominant phenomenon prior to alveolar myofibroblast activation at pulmonary parenchyma.[2,3,5] Therefore, these data suggested that continuous expansion of myofibroblasts and macrophages were not only correlated with IPF development at early stages, but also served as determinants in pathological changes of IPF at late stage.

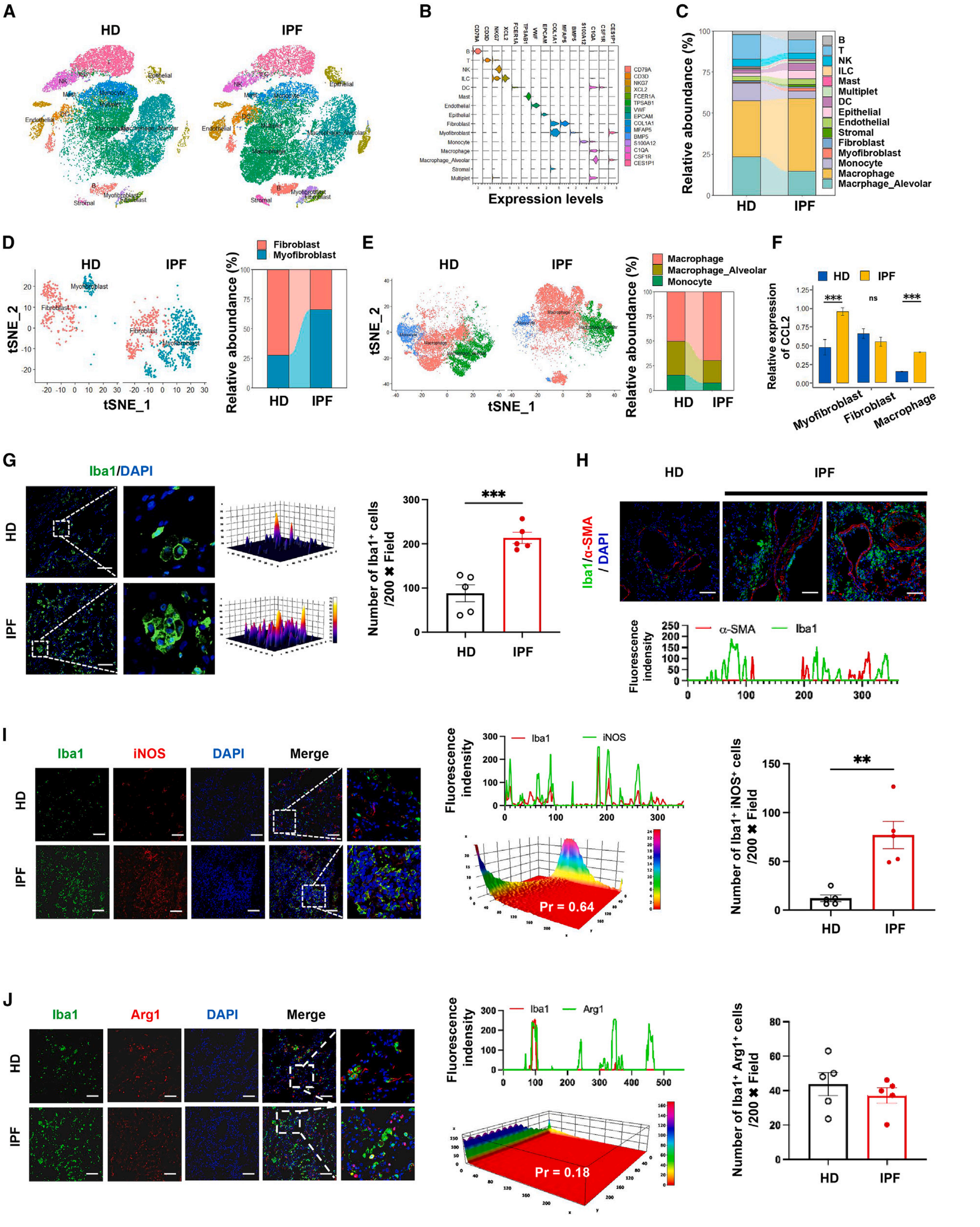

(legend on next page)

Of note, both scRNA-seq analysis and immunofluorescence (IF) revealed a significant increasing of monocyte/macrophage (F4/80, CD11b and Iba1) numbers in the lung tissue of IPF patients (Figures S4A, S4B, and S4D). By co-staining Iba1 with α-SMA, the colocalization of increasing activated myofibroblasts and macrophages at the lesion site of lung parenchyma from our involved IPF patients were further concluded (Figures 3G and 3H). In order to highlight the correlation of macrophage polarization with activating myofibroblasts, we next explored macrophage phenotypes in the lung of IPF patients and HDs. Both scRNA-seq analysis and IF results identified increased M1 macrophage (iNOS, CD86, and TNF-α) with equivalent frequencies of M2 macrophages (Arg1, CD206, and CD163) at pulmonary parenchyma of IPF patients (Fig. 3I-J and S4B-C, E-F). Indeed, in comparing with HDs, over 5-folds increasing of iNOS, CD86, and TNF-α were found in lung of patients with IPF, thus reflecting an excessive M1 macrophage polarization (Figures 3I and S4E). According to the well-documented pro-inflammatory characteristics of the M1 phenotype, our results are consistent with the finding that macrophage-mediated alveolitis is one of the driving forces for myofibroblast activation.[13,14,41] Overall, other than massive macrophage accumulation and myofibroblast activation within the lung of IPF patients, current results demonstrated that expanded M1 macrophage subset might act as a key player in facilitating IPF development.

M1 macrophages manipulated alveolar myofibroblast activation

To further dissect whether activated myofibroblast-mediated fibrosis foci formation in response to alveolitis via accumulation of M1 macrophages, fibroblast cell line NIH3T3 was employed to co-culture with induced M1 macrophages (Figures 4A and 4B). Peritoneal macrophages were purchased and following polarization by lipopolysaccharide (LPS), over 85% of them expressed iNOS and CD86, which are the well-recognized markers of M1 macrophages (Figure 4A). After 24 h of co-cultivation, peritoneal macrophages with M1 phenotype successfully enhanced NIH3T3 fibrotic cell activation due to significant expression of α-SMA (Figure 4B). Thus, accumulation of M1 macrophages at the lesion site that expresses iNOS may contribute to alveolitis in linking transition of alveolar myofibroblast to prompt fibrotic progression.

Following, in association with our above observations of IPF patients, macrophage accumulation in the pulmonary parenchyma of BI-induced murine models was detected. In line with our aspects from human IPF patients, absolute numbers of macrophages were found to largely elevate in lung tissues of BI-induced mice at day 30 and 45 (Figures 3G, 3H,4C, 4D, and S5A). Notably, those increased macrophages also co-localized with α-SMA$^+$ myofibroblasts (Figure 4E). Meanwhile, both BLM and IR groups showed comparable macrophage numbers with the control group that was different from the progressive mode of IPF patients at late stages (Figures 3G, 3H, 4C, 4D, and S5A). Next, macrophage phenotypes were detected in our IPF models. Although substantial expression of iNOS was monitored in lung resident macrophages among all groups during fibrotic change, only BI-induced mice exhibited excessive iNOS$^+$ macrophage on days 30 and 45 (Figure 4F, 4G, and S5B). Furthermore, compared with the control group, sustained expression mRNA levels of *ccr2* with increased *ccl2* and *tnfa* (typical M1 macrophagerelated pro-inflammatory cytokines), as well as quiescent *il10* (typical M2 macrophage related anti-inflammatory cytokines) in the lung tissues of BI group and IPF patients (Figures 4H, S5C, and S5D). Therefore, combined with the results regarding behavior changes and pathological characteristics during pulmonary fibrosis development, increasing M1 macrophages in lung parenchyma prompted us to believe that BI-induced murine IPF model represented most features of human patients with IPF.

M1 macrophage polarization via CCL2/CCR2 signaling

According to our trajectory analysis for IPF patients, enhanced expression of CCL2 was found in activated fibrotic cells and macrophages from IPF patients, respectively (Figure 3F and S3E–S3I). On the other hand, constitutive CCR2 expression levels were found in macrophages located in lesion sites of IPF patients (Figure S5D). Pathway enrichment analysis (Kyoto Encyclopedia of Genes and Genomes [KEGG] assay) revealed the activation of CCL2/CCR2 axis, including JAK/STAT, MAPK, and NF-κB signaling pathways, which are responsible for macrophage polarization (Figure S3J). Due to the comprehensive roles of those downstream signaling pathways in regulating macrophage migration, proliferation, and polarization, the CCL2/CCR2 axis may indeed be a promising target for controlling macrophages.[42,43] Simultaneously, consistent with the findings in human IPF patients, expression of CCL2 and CCR2 in lung tissues were positively correlated with disease progression of BI-mediated IPF murine model as well (Figures 4H and S5C). Thus, CCL2/CCR2 signaling activation was believed to result in macrophage recruitment and

Figure 3. Identification of immune cells in pulmonary parenchyma of IPF patients

(A) T-distributed stochastic neighborhood embedding (t-SNE) plots to visualize cell-type clusters based on the expression of known marker genes in lung samples of healthy donors and IPF patients. (B) Cells either from healthy donors or from IPF patients on the t-SNE plots were colored as originating. (C) Relative contributions of each cellular population identified from healthy donors and IPF lungs as shown by t-SNE. (D) Cells identified as myofibroblast and fibroblast cells by individual annotation from scRNA-seq data were clustered. Relative contributions of myofibroblasts and fibroblasts from tissues of healthy donors and IPF patients as shown by t-SNE plots. (E) Cells identified as monocyte/macrophages by individual annotation of scRNA-seq data were combined and then clustered. Relative contributions of monocyte/macrophages from pulmonary parenchyma of healthy donors and IPF patients as shown by t-SNE plots. (F) Expression levels of CCL2 in derived myofibroblasts, fibroblasts, and macrophages of HD and IPF patients. (G) Representative immunofluorescence images and quantification analysis Iba1$^+$ (green) macrophage in lung of healthy donors and IPF patients. (H) Representative immunofluorescence images of α-SMA$^+$ (red) myofibroblasts and Iba1$^+$ (green) macrophages in lung of healthy donors and IPF patients. (I and J) Representative immunofluorescence images and quantification analysis of Iba1$^+$ (green)iNOS$^+$ (red) M1 macrophage (I) and Iba1$^+$ (green)Arg1$^+$ (red) M2 macrophages (J) in lung sections of IPF patients and healthy donors. Bar, 100 μm, data (scRNA-seq, HD: n = 12, IPF patients: n = 12; IF, HD: n = 5, IPF patients: n = 5) were expressed as mean ± SEM and analyzed using Student's t test, $^*p < 0.05$, $^{**}p < 0.01$, and $^{***}p < 0.001$ between two groups.

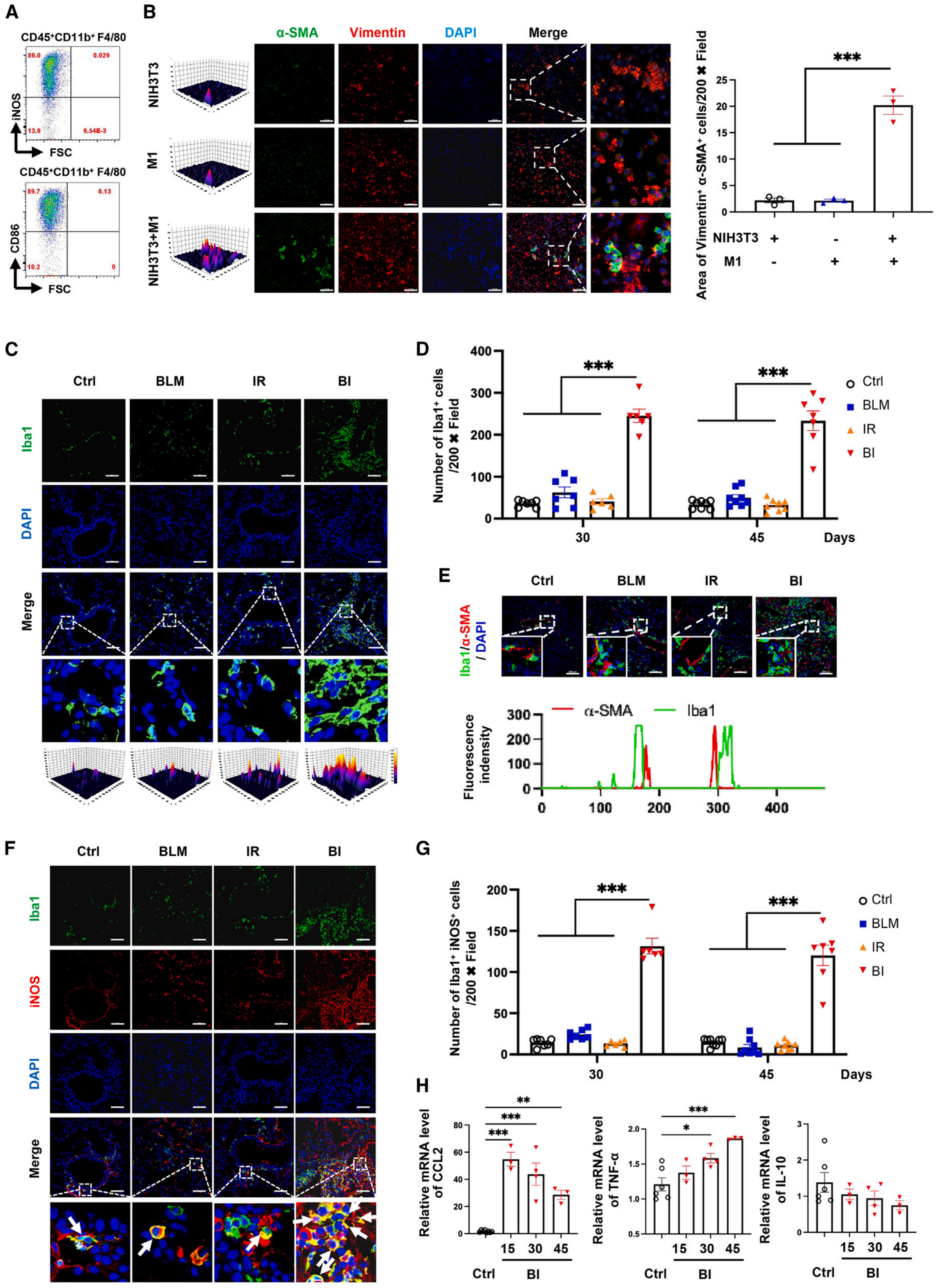

(legend on next page)

M1 phenotype polarization at pulmonary parenchyma of IPF development.

Physicochemical features of FNA-siCCR2 for CCL2/CCR2 axis

Our previous studies demonstrated the excellent targetability and accessibility of sticky-end bearing FNA in loading with siRNA to macrophages.[31–33] Here, with designated sequences to form the specific structures, multiple sticky-end bearing FNA was employed to deliver quadruple *ccr2*-siRNA that specifically interfered with the CCL2/CCR2 signaling axis in monocytes and/or macrophages (Figure 5A; Table 1). The conjunction of self-assembled FNA with quadruple *ccr2*-siRNA was confirmed by agarose electrophoresis (Figure 5B). Then dynamic light scattering (DLS) and Atomic force microscopy (AFM) were used to identify the physical features of FNA-*ccr2*-siRNA (FNA-siCCR2), including actual sizes and steric structures, respectively (Figures 5C and 5D). All synthesized FNA-siCCR2 were consistent with tetrahedral framework particles that were used in our previous studies,[32,33] and the surface character of synthesized FNA-siCCR2 was further revealed by transmission electron microscopy (TEM); accordingly, the typical pyramid structure of FNA-siCCR2 was observed (Figure 5E). Following, the efficiency of FNA-siCCR2-Cy5 endocytosis was observed in macrophages after co-incubation (Figure 5F), and over 90% of macrophages were detected to have increased median fluorescence intensity from Cy5 compared with the control group (Figure 5G). Most importantly, FNA-siCCR2 significantly inhibited the expression of CCR2 genes in macrophages (Figure S6A). To further explore the internalization of FNA-siCCR2-Cy5, we tested whether inhibitors of the clathrin and caveolae-dependent endocytosis pathway, macropinocytosis, as well as microfilaments and microtubules related to phagocytosis, respectively (Figure S6B). As shown, all inhibitors could significantly block the uptake of FNA-siCCR2-Cy5 due to the decreasing of fluorescence intensity from macrophages after 6-h incubation (Figure S6B). Meanwhile, macrophages from the Methyl-β-cyclodextrin group exhibited the most prominent decease of fluorescence intensity, which indicated that caveolin-mediated endocytic process might be the major pathway for up taking of FNA-siCCR2 (Figure S6B). After intravenous (i.v.) injection with FNA-siCCR2-Cy5 to BALB/c-nu mice, an *in vivo* imaging system was employed to continuously monitor drug distribution. The peak of fluorescence intensity from Cy5 appeared in the thoracic region at 20 min after injection, and gradually decreased to even distribution starting from 60 min after injection (Figures 5H and S7A). Notably, after tissue isolation, a large amount of nanodrug fluorescence signal could still be detected in the liver, lung, and kidney (Figure 5I). Furthermore, we employed flow cytometry to assess the cell distribution of FNA-siCCR2-Cy5 signals in PBMCs of blood and lung tissues (Figures S7B–S7D). As shown, the FNA-siCCR2-Cy5 signals were specifically localized in monocytes/macrophages, but not in B/T cells (Figures S7B–S7D). Overall, FNA-siCCR2 has convincing endocytotic efficiency, and *ccr2*-siRNA conjunction did not alter all physicochemical characterizations of FNA.

Pulmonary fibrotic progression was rescued via FNA-siCCR2 treatment in an IPF murine model

In order to generally knock down the CCL2/CCR2 axis effected on monocyte and/or macrophage migration and polarization, continuous FNA-siCCR2 administration was performed on the BI-triggered IPF murine model (Figure 6A). On day 7 after intratracheal BLM administration, irradiated mice were i.v. injected with 1 μM of FNA-siCCR2 every 2 days until day 28 post-induction (Figure 6A). Flow cytometry was employed to assess CCR2 expression in PBMCs and lung tissues. As expected, the CCR2 levels in the FNA-siCCR2 group were significantly lower than those in the PBS or FNA-scrRNA groups (Figures S7E and S7F). Moreover, in comparing with PBS-treated IPF mice, FNA-siCCR2 successfully eliminated excessive mononuclear cell accumulation in IPF mouse pulmonary parenchyma, which showed comparable macrophage numbers as control groups with or without FNA-siCCR2 treatment (Figures 6B–6D). Besides, less intense α-SMA and Vimentin expression with decreased cell numbers were found at pulmonary parenchyma in IPF mice after FNA-siCCR2 administration, but not the PBS-treated group (Figures 6E–6G). These data indicated that recruiting of monocytes and/or macrophages in polarizing to M1 phenotype could be restrained by FNA-siCCR2 and thus rescued myofibroblast activation in lung tissues of the IPF murine model.

Other than the extinguishment of immune and fibroblast aggregation, FNA-siCCR2 also successfully prevented IPF progression (Figure 6). Pathologically, MASs and area of collagen deposition were significantly reduced in the pulmonary parenchyma, which illustrated a disease remission (Figures 6H, 6I, and S7G). In line with the treatment effects in referring pathological changes, an improvement of survival rate in IPF mice was monitored after FNA-siCCR2 treatment (Figure 6J). Until day 28, FNA-siCCR2-treated IPF mice kept 100% of survival rate vs. approximately 70% of survival rate in the PBS treatment group (Figure 6J). Simultaneously, to compare with control groups, no obvious body weight loss, as well as pulmonary

Figure 4. M1 macrophage-mediated alveolar myofibroblast activation in murine models

(A) Flow cytometry results of *in vitro* M1 macrophage induction. (B) Representative immunofluorescence images and quantification analysis of NIH3T3 transmitted to α-SMA$^+$ (green)Vimentin$^+$ (red) myofibroblasts after co-culture with polarized peritoneal M1 macrophage, n = 3 for each group. (C and D) Representative immunofluorescence images and quantification analysis Iba1$^+$ (green) macrophage in lung tissues of murine models on the days 30 and 45 after induction, n = 6–8 for each group of days 30 and 45. (E) Accumulation of Iba1$^+$ (green) macrophages and α-SMA$^+$ (red) myofibroblasts at lesion site of pulmonary parenchyma from murine models. (F) Representative immunofluorescence images of Iba1$^+$ (green)iNOS$^+$ (red) M1 macrophage in lungs of murine models on the day 45 after induction. (G) The statistical analysis of Iba1$^+$ (green)iNOS$^+$ (red) M1 macrophage in lung tissues of murine models on the days 30 and 45 after induction, n = 6–8 for each group of day 30 and 45. (H) The mRNA expression levels of CCL2, TNF-α, and IL-10 in pulmonary tissues from BI group at days 15, 30, and 45 post-induction, n = 3–6 for each group. Bar, 100 μm, data were expressed as mean ± SEM and analyzed using one-way ANOVA with Tukey's multiple comparisons test, *$p < 0.05$, **$p < 0.01$, and ***$p < 0.001$ between two groups.

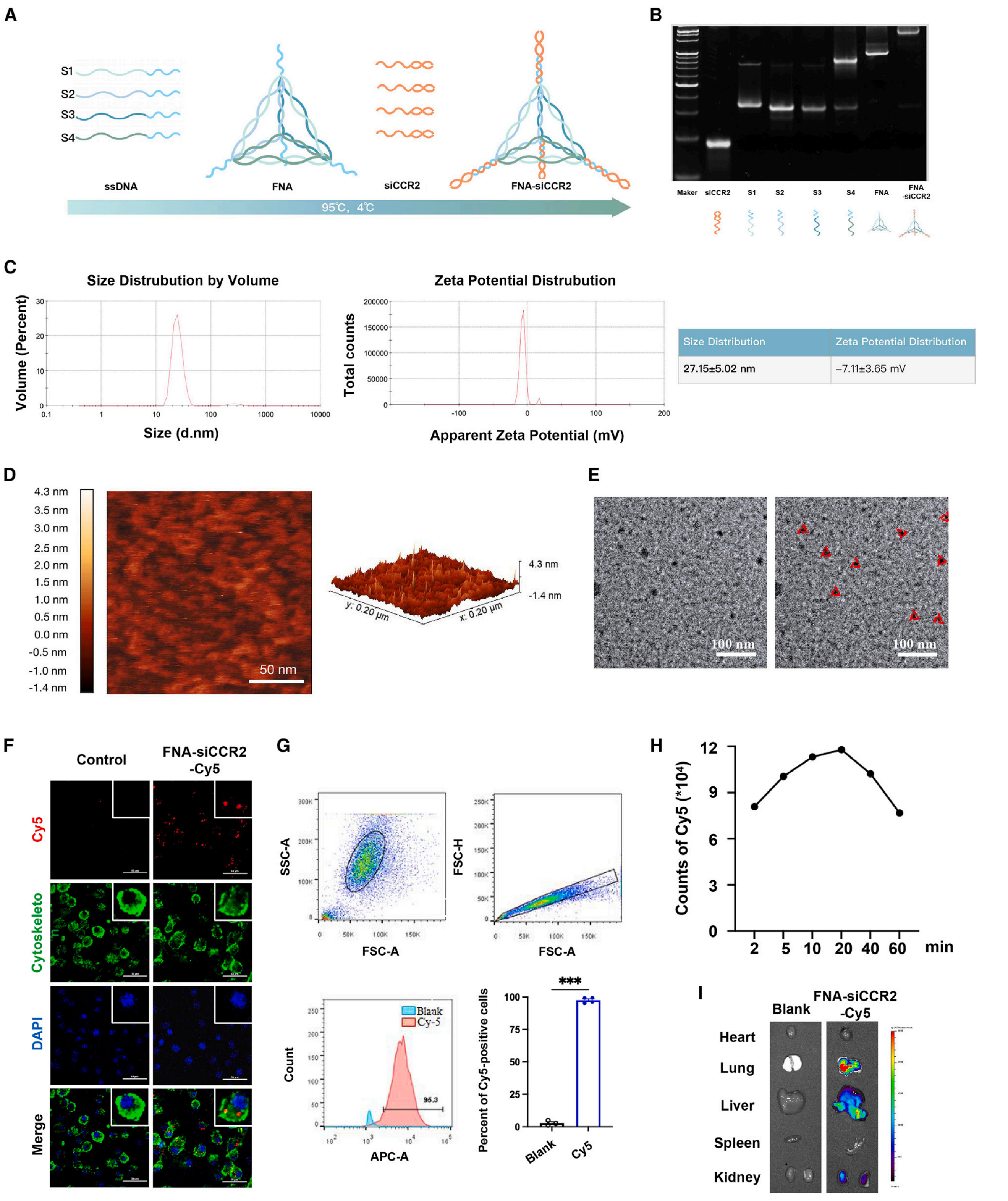

(legend on next page)

dysfunctions were detected in FNA-siCCR2-treated IPF mice (Figures 6K and 6L). Additionally, due to long-term administration, cytotoxicity of FNA-siCCR2 for metabolizing organs and circulation system was monitored, neither cytotoxic effect nor dysimmunity, including local inflammation, decreasing of resident macrophages or immune cell infiltration was observed in liver, kidney, and heart (Figure S7H). Altogether, FNA-siCCR2 efficiently blocked M1 macrophage accumulation in pulmonary parenchyma of the IPF murine model to prevent myofibroblast activation, thus leading to the disease remitting.

DISCUSSION

IPF is a chronic and progressive lethal disease that often affects populations with other pulmonary diseases, such as those with lung cancer, infectious pneumonia, and chronic obstructive pulmonary disease.[1–4] The incidence and prevalence of IPF have been rising in recent years due to population aging.[44] Other than alveolitis-mediated myofibrotic transition, imbalance of macrophage polarization is recognized to contribute to disease initiation and development, but the mechanism remains unclear.[13–15,40] By constructing and evaluating this novel chemoradiation-induced IPF murine model, we identified the similarities of clinical symptoms and pathological changes between the murine model and IPF patients. Our finding also provided evidence that M1 macrophages play pivotal roles in promoting alveolar myofibroblast activation and pulmonary fibrotic loci formation during disease progression. After determining the key effects of the CCL/CCR2 axis in macrophage accumulation and M1 polarization in the lung tissue, novel targeted delivery drug FNA-siCCR2 was synthesized to impede pulmonary fibrosis via selectively depleting M1 macrophages, thus shedding a light on the novel therapeutic strategy for IPF.

BLM triggered pulmonary fibrosis murine model is one of the essential tools in the research for IPF pathological changes.[28,29] However, the use of BLM-mediated IPF model is restricted due to self-recovery after induction.[45,46] Therefore, IR was employed in our unequivocal experimental setting for irreversible pulmonary fibrosis, which replicated most of the clinical aspects of human IPF patients. As the subtype of interstitial pneumonia, IPF exhibits irreversible progression with poor prognosis.[8,18,27] Patients with IPF mainly display severe pulmonary dysfunction, including dyspnea, hypoxemia, and hypokinesia.[4,47] Our results demonstrated that BI-induced mice mirrored motor deficiency with progressive weight loss of IPF patients. The BI-mediated IPF murine model also imitated the pathological changes in IPF patients, with characteristic alveolitis starting from the initial stage, formation of fibrotic foci at pulmonary parenchyma, as well as constitutive collagen deposition.[1,11] Additionally, as the typical signs of EMT, myofibroblast increasing and Vimentin expression suggested a potential role of fibroblast transition from alveolar epithelial cells.[48,49] Consistent with previously published reports, our findings in both human patients and the murine model emerged an *in situ* accumulation of macrophages, which contributed to connect alveolitis and mesenchymal transition for disease progression.[1–5] Although it remains elusive how the cascades of macrophage-mediated immune responses targeting lung tissues are ignited in IPF progression, other than senescence-associated pulmonary fibrosis, those above-mentioned features implied that this murine model might represent more closely to the IPF subtype sensitized by infection or environmental factors.[6,50] Another distinguishing feature of BI-induced mice as a model for human IPF is the relatively short-term disease course. Clinically, patients with IPF suffer a long-term progression, and the median survival period post-diagnosis is estimated at 2.8 years.[3,4] In BI-induced mice, a sudden onset (within 10 days) of fibrotic foci formation in pulmonary parenchyma was monitored, and it seemed to persist and remain stable. Therefore, whether severe fibrosis and the absence of overt recovery in our murine model could be attributed to acute alveolitis is an intriguing topic for future investigation. Moreover, slightly decreasing trends of collagen deposition and the number of M1 macrophages were found between day 30 and day 45, as well as a decreasing trend in CCL2 expression at the time points of 15, 30, and 45 days. Although it is not statistically significant, this stable disease might be from the remitting status of disease before secondary progression, which is one of the typical characteristics of IPF development due to the observation from patients.[51] Further studies could be drawn on the future investigations in dissecting the progression of relapsing-remitting in IPF.

According to our current results from both human and murine models, sustained alveolitis was the main effector in pulmonary fibrosis, which was initiated by macrophages through phagocytosis and/or inflammatory cytokine releasing. Over past decades, M1 macrophages are responsible for wound healing after alveolar epithelial injury, while M2 macrophages are designated to resolve the wound healing process, or terminated inflammatory responses in lung.[41,47,52] Both of them are confirmed to act as vital regulators in promoting the course of IPF development.[13,53,54] In BI-induced mice, macrophages were abundantly accumulating in fibrotic foci of symptomatic mice. The relatively high levels of iNOS expression in recruited macrophages led us to be inclined to the direct roles of M1 macrophages in triggering fibrosis at pulmonary parenchyma.

Figure 5. Characterization of FNA-siCCR2

(A) Estimated structures of FNA-siCCR2. (B) Polyacrylamide gel analysis for identification of the molecular weight of synthesized FNA-siCCR2. (C) The hydrodynamic size and zeta potential of FNA-siCCR2 detected by dynamic light scattering. (D) Atomic force microscope image of FNA-siCCR2; bar, 50 nm. (E) Representative figures of transmission electron microscope image of FNA-siCCR2; bar, 100 nm. (F) Fluorescence images of primary monocyte/macrophage after being co-cultivated with FNA-siCCR2-Cy5 (red) for 6 h; bar, 50 μm. (G) Flow cytometry results of primary monocyte/macrophage after co-cultivated with FNA-siCCR2-Cy5 for 6 h, n = 3–4 for each group. (H) Continuous observation of FNA-siCCR2-Cy5 signals in mice after i.v. injection by *in vivo* imaging system. (I) Fluorescence images of isolated organs after 60-min injection of FNA-siCCR2-Cy5, and a large amount of nanodrugs remained in the liver, lungs, and kidneys. Data were expressed as mean ± SEM and analyzed using Student's t test, ***$p < 0.001$ between two groups.

Table 1. Single-stranded DNA and RNA of FNA-siCCR2

Nucleotide strand	Direction	Base sequence
S1	5′-3′	ATTTATCACCCGCCATAGTAGACGTATCACCAGGCAGTTGAGACGAACATTCCTAAGTCTGAATTTTTATTGATCTATGATCGTACGAT
S2	5′-3′	ACATGCGAGGGTCCAATACCGACGATTACAGCTTGCTACACGATTCAGACTTAGGAATGTTCGTTTTTATTGATCTATGATCGTACGAT
S3	5′-3′	ACTACTATGGCGGGTGATAAAACGTGTAGCAAGCTGTAATCGACGGGAAGAGCATGCCCATCCTTTTTATTGATCTATGATCGTACGAT
S4	5′-3′	ACGGTATTGGACCCTCGCATGACTCAACTGCCTGGTGATACGAGGATGGGCATGCTCTTCCCGTTTTTATTGATCTATGATCGTACGAT
sense siCCR2	5′-3′	**uGcuAAAcGucucuGcAAA**dTsdT
antisense siCCR2	5′-3′	**UUUGcAGAGACGUUuAGcA**dTsdTATCGTACGATCATAGATCAAT

Bold letters represent RNA and lowercase letters represent 2′-OMe modified residues; dTsdT: dT-deoxyribonucleic thymine, s-phosphorothioate bond.

Indeed, upon alveolitis, M1 macrophages generate a pro-inflammatory microenvironment in favor of BLM-induced lung fibrosis.[55] In addition, polarized M1 macrophages may further activate through CCL/CCR2 axis, leading to excessive collagen deposition along with distorted lung tissue architecture, and ultimately resulting in pulmonary fibrosis and respiratory failure.[42,43] Simultaneously, as the specific marker that was expressed in M2 macrophages, comparable Arg1 levels in pulmonary macrophages were also found in both HDs and IPF patients. Since the functions of M2 macrophages could be regulated by a broad array of mediators, such as TNF, IFNγ, TGF-β, and IL-10, which mainly from M1 macrophages.[56] Furthermore, M2 macrophages have additional effects in activating and modulating adaptive immune responses through Ag processing/presentation and engagement in the T cell co-stimulatory pathway.[57–59] Further adaptive immunity might also participate the maintenance of alveolitis and thus facilitate the development of IPF.[13–16] The observations from clinical practice indicated that bronchoalveolar lavage fluids from IPF patients contain both M1 and M2 macrophages in some cases.[39,40] Dynamic changes of alveolar macrophages in patients with IPF at different stages were reported as well.[10–12] Therefore, we could not exclude the possibility that M2 macrophages contribute to disease progression in certain circumstances, and the underlying mechanism of how macrophage polarization effects pulmonary fibrosis is yet to be fully established. Simultaneously, except for the slight difference in CD45 expression at the early stage, both infiltrated and resident macrophages share the same features in dynamic changes of their phenotypes.[41,60] Hence, further investigation on the alternative polarization patterns of infiltrated monocytes and resident macrophages during the early stage of IPF would be an interesting issue for discovering a novel therapeutic strategy.

Current pharmacological treatment is limited and LTx is a viable option only for appropriate patients.[3] Other than treatments that reverse fibrosis, such as pirfenidone and nintedanib, studies based on the prevention of disease progression are considered to be the trends of IPF management.[61] Our results combined with other translational research have confirmed the pro-inflammatory roles of macrophages as an important component in IPF etiology, but not an epiphenomenon of fibrosis.[42,43,55] Our previous studies implicated that FNA improves the stability and delivery efficiency of RNA drugs (such as siRNA), and most recently we confirmed that the newly synthesized FNA-siRNA (third generation) with different chemical characteristics exhibits superior gene silencing efficacy and stability both *in vitro* and *in vivo*.[62–65] Thus, by employing third generation FNA, a promising biosafety nanomaterial with excellent delivery efficiency for siRNA, we targeted the CCL/CCR2 axis to eliminate macrophage accumulation in the IPF murine model.[42,43,65,66] BI-induced pulmonary fibrosis was attenuated after FNA-siCCR2 administration, which mainly effected on macrophages decreasing in pulmonary parenchyma. Due to less specificity of FNA in targeting designated tissues, the systemic delivery of FNA-siCCR2 could not be fully accumulated in lung. This insufficient targetability might weaken the response rate and bring unexpected adverse events during treatment. In subsequent studies, we will further improve the tissue targetability of FNA-siCCR2. Based on our previous investigation in anti-fibrotic and anti-inflammatory effects of FNA, we could not exclude the possibilities that FNA also directly reduces pulmonary fibrosis via suppressing TGF-β1/Smads-mediated EMT and/or systemic inflammation.[32] Meanwhile, with the general decreasing of pulmonary macrophage numbers, we speculated that the CCL/CCR2 axis might be responsible for macrophage proliferation as well.[42,43] In addition, despite the potential downstream signaling of CCR2 from Gene Ontology (GO) analysis, detailed information regarding imbalance of macrophage polarization from CCL2/CCR2 axis blockade should be further identified.

Overall, by constructing a novel IPF animal model, our studies lay the groundwork for understanding M1 macrophage-mediated cellular immune responses in IPF pathogenesis in both human and murine models. These findings are highly relevant to many clinical scenarios and translational research. The establishment of FNA-siCCR2 treatment in targeting macrophages also provides an accessible way for preventing pulmonary fibrosis and IPF.

MATERIALS AND METHODS

Animals

All animals were treated according to the experimental procedure approved by the Institutional Animal Care and Use Committee (IACUC) of Sichuan Cancer Hospital, following the guidelines of the

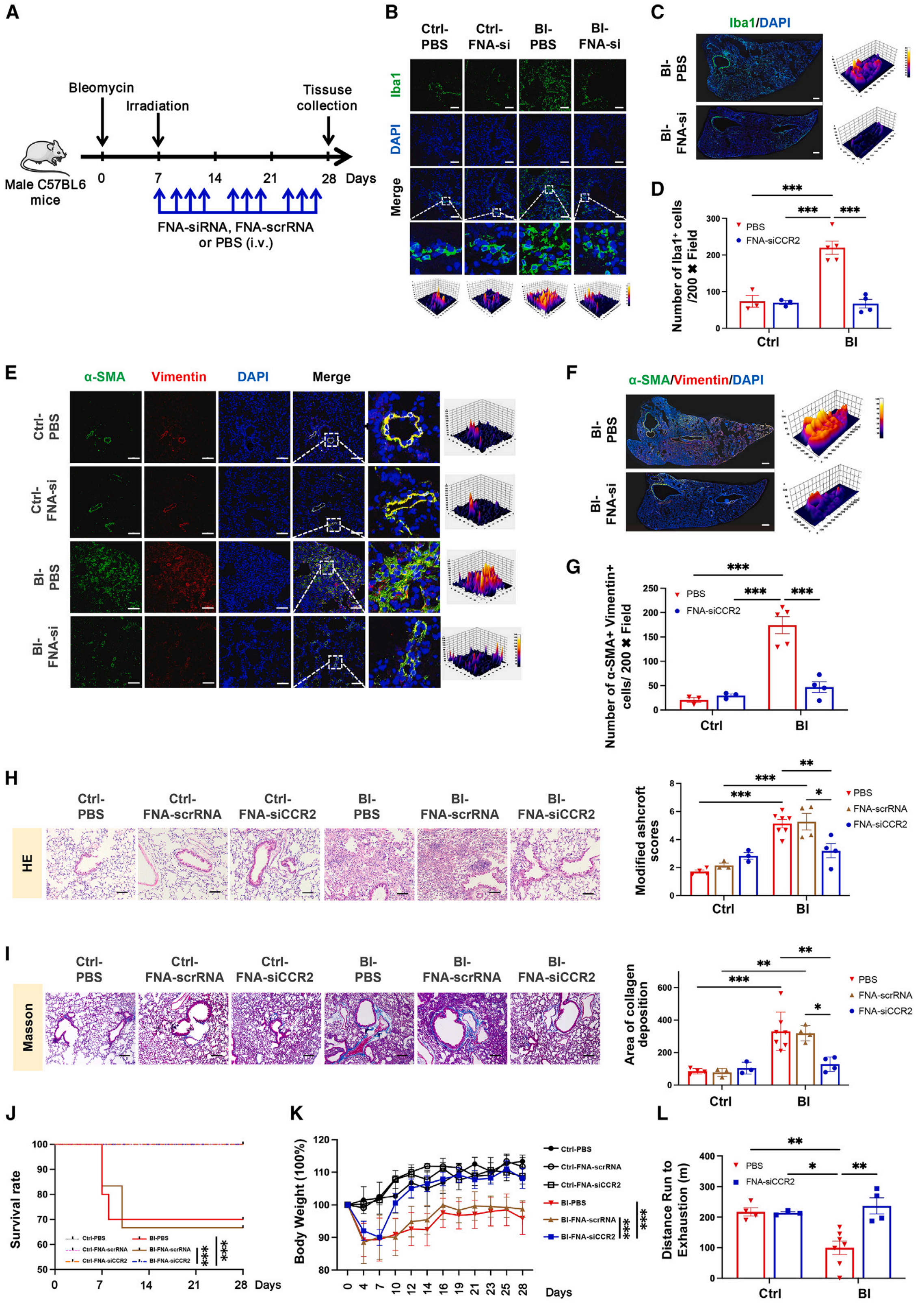

(legend on next page)

National Institutes of Health (NIH) (approval number/ID: SCCHEC-04-2023-001). Male C57BL6 mice (HuaFuKang), aged 7–8 weeks old, were housed in individually ventilated cages under standard laboratory conditions (22 ± 2°C, humidity 40%–70%, 12 h dark/light cycles, pathogen-free) with food and water *ad libitum*. The mice were accustomed to the conditions for at least 1 week in the Animal Care and Use Center. Animal pain and stress was minimized throughout the study.

Human lung specimens

Research procedures were performed following the standards of the Ethical for Biomedical Research Involving Humans and the Ethics Committee for Medical Research of Sichuan Provincial People's Hospital, University of Electronic Science and Technology of China (approval number/ID: 2023-29). All the patients were included according to the 2018 ATS/ERS/JRS/ALAT Clinical Practice Guideline of IPF[4] and met the selection criteria and did not have significant contraindications for lung transplant.[67] Signed informed consent was obtained to procure lung specimens from individuals diagnosed with IPF at Sichuan Provincial People's Hospital. Lung tissues were collected from five IPF patients, who received LTx (Table S1), and five paired lung tissues of HDs were also acquired at the same time. The samples were fixed in 4% paraformaldehyde (PFA) and then sectioned to thickness for histopathological analysis.

Bleomycin and X-irradiation treatment

Following anesthesia, 2.5 U/kg BLM (CSNpharm, CSN10472) was intratracheally administrated to the lung, and then a single dose of 10 Gy IR was performed on the whole thorax after 1 week. Briefly, mice were subjected to endotracheal intubation after anesthesia with pentobarbital sodium (45 mg/kg) by intraperitoneal injection. Sterile saline (50 μL) with or without BLM was pipetted into the endotracheal cannula as in the previous studies. After a week, anesthetized mice were placed in the supine position, and a single dose of 10 Gy X-ray irradiation was delivered using a biological radiometer (Precision X-ray, American) only to the chest region with the remainder of the body covered with a 5-mm lead plate.

Synthesis and characterization of FNA-siCCR2

The FNA was synthesized by four sticky-end modified single-stranded DNAs (sS1-sS4) (Sangon) (Table 1). Briefly, the equimolar quantities of sS1-sS4 were denatured at 95°C for 10 min, and then cooled to 4°C for 30 min. After the fabrication of FNA, siCCR2 was connected to the FNA through the complementary pairing in a ratio of 4:1 at room temperature for 20 min. Characterization of FNA-siCCR2 was performed using polyacrylamide gel analysis, DLS, AFM, and TEM.

In vitro cellular uptake of FNA-siCCR2

Primary monocyte/macrophage was harvested from the femur and tibia of C57BL6 mice. The obtained cells were cultured in DMEM with 10% FBS (Gibco), 1% penicillin-streptomycin (HyClone), and 20 ng/mL M-CSF (Novoprotein) at 37°C with 5% CO_2. After 6 days, cells were treated with 250 nM Cy5-labed FNA-siCCR2 for 6 h. Immunofluorescence and flow cytometry were performed to demonstrate the cellular uptake of FNA-siCCR2-Cy5.

Live animal imaging system

After being anesthetized with 2% isoflurane, BALB/c-nu mice were injected with 100 μL FNA-siCCR2-Cy5 via a tail vein. Using a small animal imaging system (IVIS Spectrum; Caliper Life Sciences), images of the fluorescence signal were obtained after 2–60 min. Simultaneously, the drug in the tube was placed aside as a positive control.

Histopathological analysis and immunofluorescence staining

The mice were killed systematically to harvest lung tissues at different time points (7, 15, 30, and 45 days after the intervention). Left posterior lobe of lung tissues in mice and three different areas of lung tissue in IPF patients were collected and fixed in 4% PFA, embedded in paraffin, and sectioned at 5-μm thickness. The paraffin sections were gradually stained with hematoxylin-eosin (H&E). Total collagen was histologically assessed by both Sirius Red and Masson's Trichrome staining. Three to five visual fields of each sample were randomly selected and collected with a Nikon Eclipse 80i microscope connected to a video camera and analyzed by ImageJ software to evaluate MAS and collagen deposition. Other lung tissues were incubated in 30% sucrose overnight after 4% PFA treatment, and then the tissues were embedded in OCT and sectioned at 4-μm thickness for immunostaining experiments. Briefly, the slices were blocked with 3% BSA for 1 h, and then were incubated gradually with primary antibodies (rabbit anti-Vimentin [1:400], mouse anti-α-SMA [1:400], rabbit anti-Iba1 [1:400], mouse anti-iNOS [1:200], mouse anti-Arginase1 [1:200]) at 4°C overnight, an Alexa

Figure 6. FNA-siCCR2 prevent pulmonary fibrosis in IPF murine model

(A) Chemoradiation-induction of IPF and treatment strategies. (B) Representative immunofluorescence images of $Iba1^+$ (green) macrophage in lung tissues of murine IPF model after FNA-siCCR2 treatment; bar, 100 μm. (C) The general scan of $Iba1^+$ (green) macrophage in lung tissues from murine IPF model after FNA-siCCR2 treatment; bar, 500 μm. (D) Quantification analysis of $Iba1^+$ (green) macrophage in lung tissues of murine IPF model after FNA-siCCR2 treatment, n = 3–5 for each group. (E) Representative immunofluorescence images of α-SMA^+ (green)$Vimentin^+$ (red) myofibroblasts in lung tissues of murine IPF models after FNA-siCCR2 treatment; bar, 100 μm. (F) The general scan of α-SMA^+ (green)$Vimentin^+$ (red) myofibroblasts in pulmonary tissues from murine IPF model; bar, 500 μm. (G) Quantification analysis of α-SMA^+ (green)$Vimentin^+$ (red) myofibroblasts in lung tissues of murine IPF models after FNA-siCCR2 treatment, n = 3–5 for each group. (H) The representative images of H&E staining and statistical analysis of MAS in lung tissues from murine IPF models after FNA-siCCR2 treatment; bar, 100 μm, n = 3–7 for each group. (I) The representative images of Masson staining and statistical analysis of collagen deposition in lung tissues from murine IPF models after FNA-siCCR2 treatment; bar, 200 μm, n = 3–7 for each group. (J) Survival curve of mice, Ctrl-PBS: n = 4; Ctrl-FNA-scrRNA: n = 3; Ctrl-FNA-siCCR2: n = 3; BI-PBS: n = 10; BI-FNA-scrRNA: n = 6; BI-FNA-siCCR2: n = 4. (K) Continuous observation of body weight changes of murine models after treatment with FNA-siCCR2, Ctrl-PBS: n = 4; Ctrl-FNA-scrRNA: n = 3; Ctrl-FNA-siCCR2: n = 3; BI-PBS: n = 7; BI-FNA-scrRNA: n = 4; BI-FNA-siCCR2: n = 4. (L) The evaluation of maximal endurance running capacity at day 28 after induction, n = 3–7 for each group. Data were expressed as mean ± SEM and analyzed using two-way ANOVA with Tukey's multiple comparisons test, $^*p < 0.05$, $^{**}p < 0.01$, and $^{***}p < 0.001$ between two groups.

Fluor-conjugated secondary antibody at 37°C for 1 h, and DAPI for nuclei at room temperature for 30 min. Each photograph of the stained sections was collected with a confocal microscope and analyzed by ImageJ software.

Measuring exercise capacity

Mice were trained to be familiar with the treadmill and thus were adapted to the experimental environment for 7 days before data collection. The maximal endurance running capacity test of mice was evaluated at different time points after establishment of the IPF model or drug treatment. Briefly, mice performed on a runway of 15° slope with the starting velocity at 10 m/min and increasing by 1 m/min until the mice could no longer keep pace with the treadmill. Each experiment was repeated in triplicate and the average value of distance was recorded as the final endurance running distance of the mouse.

Single-cell RNA-seq analysis

The lung sample (n = 1) from an IPF patient was harvested and dissociated into single cell suspensions by mixed digestion buffer. Cells were collected after filtering through a 70-μm and 40-μm strainer. The viability and concentration of cells were calculated in a Counting Star system. Single-cell suspensions were loaded on the Chromium Single Cell Controller (10X genomics) for analysis, following the manufacturer's instructions. Lung samples from the GEO database (Database: GSE136831, GSE122960) were used for the combined data analysis.[68,69] The scaled expression datasets were clustered using a graph-based clustering algorithm (implemented in the "Seurat" package, v4.0.6) and were presented using t-SNE plots. The batch effect was adjusted using *harmony* (v0.1.0). Identities of clusters were manually annotated using well-recognized cell markers according to published articles. Pseudotime trajectory analysis was performed using the *Monocle2* package (v2.14.0) based on the DDRTree method. Deep analysis, including Gene Ontology (GO) enrichment analysis and Kyoto Encyclopedia of Genes and Genomes (KEGG) pathway enrichment analysis, was also further performed.

Statistical analysis

Results represent the mean ± SEM. Statistical significance was determined as indicated in figure legends. Student's t test was used to compute statistical significance between two groups. One-way ANOVA with Tukey's multiple comparisons test was used to compute statistical significance between multiple groups. The comparisons between groups were made using two-way ANOVA with Tukey's multiple comparisons test. The values of $^{*}p < 0.05$, $^{**}p < 0.01$, and $^{***}p < 0.001$ were considered as statistically significant.

DATA AND CODE AVAILABILITY

Data, further information, and requests for resources and reagents supporting the findings of this study are available from the corresponding author Mu Yang (mu.yang@uestc.edu.cn). The datasets presented in the study can be found in the Gene Expression Omnibus (GEO) Database: GSE253348.

SUPPLEMENTAL INFORMATION

Supplemental information can be found online at https://doi.org/10.1016/j.ymthe.2024.01.022.

ACKNOWLEDGMENTS

This study was supported by grants from the Medicine and Engineering Cross-Innovation on Oncology of University of Electronic Science and Technology of China (ZYGX2021YGCX005) to M.Y.; the National Natural Science Foundation of China (82300276) to C.L.; the Sichuan Postdoctoral Foundation (TB2022085) to C.L.; the Sichuan Natural Science Foundation (2023NSFSC1643, 2023NSFSC1672, 2023ZYD0045) to C.L., J.Y., and M.Y.; the Excellent Youth Fund of Sichuan Cancer Hospital (YB2024017) to X.F.; and the Sichuan Young Talents Foundation to M.Y.

AUTHOR CONTRIBUTIONS

M.Y., L.G., and Y.L. were involved in conceptualization; C.L., X.F., X.C., X.W., and J.Y. performed the experiments, analyzed data, and wrote the manuscript; S.L. and X.C. performed the synthesis of tetrahedral framework nucleic acids; X.H. and L.G. provided tissue specimens and clinical information; Z.L., J.X., D.W., D.L., H.Z., and L.M. provided technical support; M.Y., C.L., and J.Y. were involved in funding acquisition; C.L. and X.F. wrote the original draft; M.Y., L.G., Y.L., and T.L. reviewed and edited the writing. All authors read and approved the final article.

DECLARATION OF INTERESTS

The authors declare no competing interests.

REFERENCES

1. Martinez, F.J., Collard, H.R., Pardo, A., Raghu, G., Richeldi, L., Selman, M., Swigris, J.J., Taniguchi, H., and Wells, A.U. (2017). Idiopathic pulmonary fibrosis. Nat. Rev. Dis. Primers *3*, 17074.
2. Moss, B.J., Ryter, S.W., and Rosas, I.O. (2022). Pathogenic Mechanisms Underlying Idiopathic Pulmonary Fibrosis. Annu. Rev. Pathol. *17*, 515–546.
3. George, P.M., Patterson, C.M., Reed, A.K., and Thillai, M. (2019). Lung transplantation for idiopathic pulmonary fibrosis. The Lancet. Lancet Respir. Med. *7*, 271–282.
4. Raghu, G., Remy-Jardin, M., Myers, J.L., Richeldi, L., Ryerson, C.J., Lederer, D.J., Behr, J., Cottin, V., Danoff, S.K., Morell, F., et al. (2018). Diagnosis of Idiopathic Pulmonary Fibrosis. An Official ATS/ERS/JRS/ALAT Clinical Practice Guideline. Am. J. Respir. Crit. Care Med. *198*, e44–e68.
5. Wolters, P.J., Blackwell, T.S., Eickelberg, O., Loyd, J.E., Kaminski, N., Jenkins, G., Maher, T.M., Molina-Molina, M., Noble, P.W., Raghu, G., et al. (2018). Time for a change: is idiopathic pulmonary fibrosis still idiopathic and only fibrotic? Lancet Respir. Med. *6*, 154–160.
6. Wendisch, D., Dietrich, O., Mari, T., von Stillfried, S., Ibarra, I.L., Mittermaier, M., Mache, C., Chua, R.L., Knoll, R., Timm, S., et al. (2021). SARS-CoV-2 infection triggers profibrotic macrophage responses and lung fibrosis. Cell *184*, 6243–6261.e27.
7. Hatabu, H., Kaye, K.M., and Christiani, D.C. (2023). Viral Infection, Pulmonary Fibrosis, and Long COVID. Am. J. Respir. Crit. Care Med. *207*, 647–649.
8. Hanania, A.N., Mainwaring, W., Ghebre, Y.T., Hanania, N.A., and Ludwig, M. (2019). Radiation-Induced Lung Injury: Assessment and Management. Chest *156*, 150–162.
9. Furusawa, H., Peljto, A.L., Walts, A.D., Cardwell, J., Molyneaux, P.L., Lee, J.S., Fernández Pérez, E.R., Wolters, P.J., Yang, I.V., and Schwartz, D.A. (2022). Common idiopathic pulmonary fibrosis risk variants are associated with hypersensitivity pneumonitis. Thorax *77*, 508–510.

10. Larson-Casey, J.L., Deshane, J.S., Ryan, A.J., Thannickal, V.J., and Carter, A.B. (2016). Macrophage Akt1 Kinase-Mediated Mitophagy Modulates Apoptosis Resistance and Pulmonary Fibrosis. Immunity *44*, 582–596.

11. Shenderov, K., Collins, S.L., Powell, J.D., and Horton, M.R. (2021). Immune dysregulation as a driver of idiopathic pulmonary fibrosis. J. Clin. Invest. *131*, e143226.

12. Yao, C., Guan, X., Carraro, G., Parimon, T., Liu, X., Huang, G., Mulay, A., Soukiasian, H.J., David, G., Weigt, S.S., et al. (2021). Senescence of Alveolar Type 2 Cells Drives Progressive Pulmonary Fibrosis. Am. J. Respir. Crit. Care Med. *203*, 707–717.

13. Lee, J.W., Chun, W., Lee, H.J., Min, J.H., Kim, S.M., Seo, J.Y., Ahn, K.S., and Oh, S.R. (2021). The Role of Macrophages in the Development of Acute and Chronic Inflammatory Lung Diseases. Cells *10*, 897.

14. Ogger, P.P., Albers, G.J., Hewitt, R.J., O'Sullivan, B.J., Powell, J.E., Calamita, E., Ghai, P., Walker, S.A., McErlean, P., Saunders, P., et al. (2020). Itaconate controls the severity of pulmonary fibrosis. Sci. Immunol. *5*, eabc1884.

15. Aran, D., Looney, A.P., Liu, L., Wu, E., Fong, V., Hsu, A., Chak, S., Naikawadi, R.P., Wolters, P.J., Abate, A.R., et al. (2019). Reference-based analysis of lung single-cell sequencing reveals a transitional profibrotic macrophage. Nat. Immunol. *20*, 163–172.

16. Weiskirchen, R., Weiskirchen, S., and Tacke, F. (2019). Organ and tissue fibrosis: Molecular signals, cellular mechanisms and translational implications. Mol. Aspects Med. *65*, 2–15.

17. Cheng, P., Li, S., and Chen, H. (2021). Macrophages in Lung Injury, Repair, and Fibrosis. Cells *10*, 436.

18. Cinar, R., Park, J.K., Zawatsky, C.N., Coffey, N.J., Bodine, S.P., Abdalla, J., Yokoyama, T., Jourdan, T., Jay, L., Zuo, M.X.G., et al. (2021). CB1 R and iNOS are distinct players promoting pulmonary fibrosis in Hermansky-Pudlak syndrome. Clin. Transl. Med. *11*, e471.

19. Whyte, M., Hubbard, R., Meliconi, R., Whidborne, M., Eaton, V., Bingle, C., Timms, J., Duff, G., Facchini, A., Pacilli, A., et al. (2000). Increased risk of fibrosing alveolitis associated with interleukin-1 receptor antagonist and tumor necrosis factor-alpha gene polymorphisms. Am. J. Respir. Crit. Care Med. *162*, 755–758.

20. Tian, Y., Lv, J., Su, Z., Wu, T., Li, X., Hu, X., Zhang, J., and Wu, L. (2021). LRRK2 plays essential roles in maintaining lung homeostasis and preventing the development of pulmonary fibrosis. Proc. Natl. Acad. Sci. USA. *118*, e2106685118.

21. Osafo-Addo, A.D., and Herzog, E.L. (2017). CCL2 and T cells in pulmonary fibrosis: an old player gets a new role. Thorax *72*, 967–968.

22. Ouyang, W., Rutz, S., Crellin, N.K., Valdez, P.A., and Hymowitz, S.G. (2011). Regulation and functions of the IL-10 family of cytokines in inflammation and disease. Annu. Rev. Immunol. *29*, 71–109.

23. Rao, L.Z., Wang, Y., Zhang, L., Wu, G., Zhang, L., Wang, F.X., Chen, L.M., Sun, F., Jia, S., Zhang, S., et al. (2021). IL-24 deficiency protects mice against bleomycin-induced pulmonary fibrosis by repressing IL-4-induced M2 program in macrophages. Cell Death Differ. *28*, 1270–1283.

24. Gao, S., Li, X., Jiang, Q., Liang, Q., Zhang, F., Li, S., Zhang, R., Luan, J., Zhu, J., Gu, X., et al. (2022). PKM2 promotes pulmonary fibrosis by stabilizing TGF-β1 receptor I and enhancing TGF-β1 signaling. Sci. Adv. *8*, eabo0987.

25. Spagnolo, P., Kropski, J.A., Jones, M.G., Lee, J.S., Rossi, G., Karampitsakos, T., Maher, T.M., Tzouvelekis, A., and Ryerson, C.J. (2021). Idiopathic pulmonary fibrosis: Disease mechanisms and drug development. Pharmacol. Ther. *222*, 107798.

26. Bickelhaupt, S., Erbel, C., Timke, C., Wirkner, U., Dadrich, M., Flechsig, P., Tietz, A., Pföhler, J., Gross, W., Peschke, P., et al. (2017). Effects of CTGF Blockade on Attenuation and Reversal of Radiation-Induced Pulmonary Fibrosis. J. Natl. Cancer Inst. *109*.

27. Gross, N.J. (1977). Pulmonary effects of radiation therapy. Ann. Intern. Med. *86*, 81–92.

28. Lazo, J.S., Hoyt, D.G., Sebti, S.M., and Pitt, B.R. (1990). Bleomycin: a pharmacologic tool in the study of the pathogenesis of interstitial pulmonary fibrosis. Pharmacol. Ther. *47*, 347–358.

29. Kolb, P., Upagupta, C., Vierhout, M., Ayaub, E., Bellaye, P.S., Gauldie, J., Shimbori, C., Inman, M., Ask, K., and Kolb, M.R.J. (2020). The importance of interventional timing in the bleomycin model of pulmonary fibrosis. Eur. Respir. J. *55*, 1901105.

30. Wu, H., Yu, Y., Huang, H., Hu, Y., Fu, S., Wang, Z., Shi, M., Zhao, X., Yuan, J., Li, J., et al. (2020). Progressive Pulmonary Fibrosis Is Caused by Elevated Mechanical Tension on Alveolar Stem Cells. Cell *180*, 107–121.e17.

31. Qin, X., Xiao, L., Li, N., Hou, C., Li, W., Li, J., Yan, N., and Lin, Y. (2022). Tetrahedral framework nucleic acids-based delivery of microRNA-155 inhibits choroidal neovascularization by regulating the polarization of macrophages. Bioact. Mater. *14*, 134–144.

32. Zhang, T., Tian, T., and Lin, Y. (2022). Functionalizing Framework Nucleic-Acid-Based Nanostructures for Biomedical Application. Adv. Mater. *34*, e2107820.

33. Li, S., Liu, Y., Zhang, T., Lin, S., Shi, S., He, J., Xie, Y., Cai, X., Tian, T., and Lin, Y. (2022). A Tetrahedral Framework DNA-Based Bioswitchable miRNA Inhibitor Delivery System: Application to Skin Anti-Aging. Adv. Mater. *34*, e2204287.

34. Zhang, R., Jing, W., Chen, C., Zhang, S., Abdalla, M., Sun, P., Wang, G., You, W., Yang, Z., Zhang, J., et al. (2022). Inhaled mRNA Nanoformulation with Biogenic Ribosomal Protein Reverses Established Pulmonary Fibrosis in a Bleomycin-Induced Murine Model. Adv. Mater. *34*, e2107506.

35. Liu, G., Philp, A.M., Corte, T., Travis, M.A., Schilter, H., Hansbro, N.G., Burns, C.J., Eapen, M.S., Sohal, S.S., Burgess, J.K., and Hansbro, P.M. (2021). Therapeutic targets in lung tissue remodelling and fibrosis. Pharmacol. Ther. *225*, 107839.

36. King, T.E., Jr., Pardo, A., and Selman, M. (2011). Idiopathic pulmonary fibrosis. Lancet *378*, 1949–1961.

37. Afratis, N.A., Klepfish, M., Karamanos, N.K., and Sagi, I. (2018). The apparent competitive action of ECM proteases and cross-linking enzymes during fibrosis: Applications to drug discovery. Adv. Drug Deliv. Rev. *129*, 4–15.

38. DeMaio, L., Buckley, S.T., Krishnaveni, M.S., Flodby, P., Dubourd, M., Banfalvi, A., Xing, Y., Ehrhardt, C., Minoo, P., Zhou, B., et al. (2012). Ligand-independent transforming growth factor-β type I receptor signalling mediates type I collagen-induced epithelial-mesenchymal transition. J. Pathol. *226*, 633–644.

39. Meyer, K.C., Raghu, G., Baughman, R.P., Brown, K.K., Costabel, U., du Bois, R.M., Drent, M., Haslam, P.L., Kim, D.S., Nagai, S., et al. (2012). An official American Thoracic Society clinical practice guideline: the clinical utility of bronchoalveolar lavage cellular analysis in interstitial lung disease. Am. J. Respir. Crit. Care Med. *185*, 1004–1014.

40. Morse, C., Tabib, T., Sembrat, J., Buschur, K.L., Bittar, H.T., Valenzi, E., Jiang, Y., Kass, D.J., Gibson, K., Chen, W., et al. (2019). Proliferating SPP1/MERTK-expressing macrophages in idiopathic pulmonary fibrosis. Eur. Respir. J. *54*, 1802441.

41. Xie, C., Wan, L., Li, C., Feng, Y., and Kang, Y.J. (2021). Selective suppression of M1 macrophages is involved in zinc inhibition of liver fibrosis in mice. J. Nutr. Biochem. *97*, 108802.

42. Brody, S.L., Gunsten, S.P., Luehmann, H.P., Sultan, D.H., Hoelscher, M., Heo, G.S., Pan, J., Koenitzer, J.R., Lee, E.C., Huang, T., et al. (2021). Chemokine Receptor 2-targeted Molecular Imaging in Pulmonary Fibrosis. A Clinical Trial. Am. J. Respir. Crit. Care Med. *203*, 78–89.

43. Raghu, H., Lepus, C.M., Wang, Q., Wong, H.H., Lingampalli, N., Oliviero, F., Punzi, L., Giori, N.J., Goodman, S.B., Chu, C.R., et al. (2017). CCL2/CCR2, but not CCL5/CCR5, mediates monocyte recruitment, inflammation and cartilage destruction in osteoarthritis. Ann. Rheum. Dis. *76*, 914–922.

44. Mora, A.L., Rojas, M., Pardo, A., and Selman, M. (2017). Emerging therapies for idiopathic pulmonary fibrosis, a progressive age-related disease. Nat. Rev. Drug Discov. *16*, 755–772.

45. Xia, H., Gilbertsen, A., Herrera, J., Racila, E., Smith, K., Peterson, M., Griffin, T., Benyumov, A., Yang, L., Bitterman, P.B., and Henke, C.A. (2017). Calcium-binding protein S100A4 confers mesenchymal progenitor cell fibrogenicity in idiopathic pulmonary fibrosis. J. Clin. Invest. *127*, 2586–2597.

46. Borzone, G., Moreno, R., Urrea, R., Meneses, M., Oyarzún, M., and Lisboa, C. (2001). Bleomycin-induced chronic lung damage does not resemble human idiopathic pulmonary fibrosis. Am. J. Respir. Crit. Care Med. *163*, 1648–1653.

47. Agarwala, P., and Salzman, S.H. (2020). Six-Minute Walk Test: Clinical Role, Technique, Coding, and Reimbursement. Chest *157*, 603–611.

48. Kim, K.K., Wei, Y., Szekeres, C., Kugler, M.C., Wolters, P.J., Hill, M.L., Frank, J.A., Brumwell, A.N., Wheeler, S.E., Kreidberg, J.A., and Chapman, H.A. (2009).

Epithelial cell alpha3beta1 integrin links beta-catenin and Smad signaling to promote myofibroblast formation and pulmonary fibrosis. J. Clin. Invest. *119*, 213–224.

49. Phan, T.H.G., Paliogiannis, P., Nasrallah, G.K., Giordo, R., Eid, A.H., Fois, A.G., Zinellu, A., Mangoni, A.A., and Pintus, G. (2021). Emerging cellular and molecular determinants of idiopathic pulmonary fibrosis. Cell. Mol. Life Sci. *78*, 2031–2057.

50. Cui, F., Sun, Y., Xie, J., Li, D., Wu, M., Song, L., Hu, Y., and Tian, Y. (2023). Air pollutants, genetic susceptibility and risk of incident idiopathic pulmonary fibrosis. Eur. Respir. J. *61*, 2200777.

51. Ryerson, C.J., Cottin, V., Brown, K.K., and Collard, H.R. (2015). Acute exacerbation of idiopathic pulmonary fibrosis: shifting the paradigm. Eur. Respir. J. *46*, 512–520.

52. Chang, X., Xing, L., Wang, Y., Zhou, T.J., Shen, L.J., and Jiang, H.L. (2020). Nanoengineered immunosuppressive therapeutics modulating M1/M2 macrophages into the balanced status for enhanced idiopathic pulmonary fibrosis therapy. Nanoscale *12*, 8664–8678.

53. Byrne, A.J., Maher, T.M., and Lloyd, C.M. (2016). Pulmonary Macrophages: A New Therapeutic Pathway in Fibrosing Lung Disease? Trends Mol. Med. *22*, 303–316.

54. Minutti, C.M., Jackson-Jones, L.H., García-Fojeda, B., Knipper, J.A., Sutherland, T.E., Logan, N., Ringqvist, E., Guillamat-Prats, R., Ferenbach, D.A., Artigas, A., et al. (2017). Local amplifiers of IL-4Rα-mediated macrophage activation promote repair in lung and liver. Science (New York, N.Y.) *356*, 1076–1080.

55. Cui, Z., Liao, J., Cheong, N., Longoria, C., Cao, G., DeLisser, H.M., and Savani, R.C. (2019). The Receptor for Hyaluronan-Mediated Motility (CD168) promotes inflammation and fibrosis after acute lung injury. Matrix Biol. *78–79*, 255–271.

56. Chen, S., Kammerl, I.E., Vosyka, O., Baumann, T., Yu, Y., Wu, Y., Irmler, M., Overkleeft, H.S., Beckers, J., Eickelberg, O., et al. (2016). Immunoproteasome dysfunction augments alternative polarization of alveolar macrophages. Cell Death Differ. *23*, 1026–1037.

57. Schroeder, B.A., LaFranzo, N.A., LaFleur, B.J., Gittelman, R.M., Vignali, M., Zhang, S., Flanagan, K.C., Rytlewski, J., Riolobos, L., Schulte, B.C., et al. (2021). CD4+ T cell and M2 macrophage infiltration predict dedifferentiated liposarcoma patient outcomes. J. Immunother. Cancer *9*, e002812.

58. Allavena, P., Sica, A., Garlanda, C., and Mantovani, A. (2008). The Yin-Yang of tumor-associated macrophages in neoplastic progression and immune surveillance. Immunol. Rev. *222*, 155–161.

59. Jaynes, J.M., Sable, R., Ronzetti, M., Bautista, W., Knotts, Z., Abisoye-Ogunniyan, A., Li, D., Calvo, R., Dashnyam, M., Singh, A., et al. (2020). Mannose receptor (CD206) activation in tumor-associated macrophages enhances adaptive and innate antitumor immune responses. Sci. Transl. Med. *12*, eaax6337.

60. Ji, X., Cai, J., Liang, L., Shi, T., and Liu, J. (2023). Gene expression variability across cells and species shapes the relationship between renal resident macrophages and infiltrated macrophages. BMC bioinformatics *24*, 72.

61. Flaherty, K.R., Fell, C.D., Huggins, J.T., Nunes, H., Sussman, R., Valenzuela, C., Petzinger, U., Stauffer, J.L., Gilberg, F., Bengus, M., and Wijsenbeek, M. (2018). Safety of nintedanib added to pirfenidone treatment for idiopathic pulmonary fibrosis. Eur. Respir. J. *52*, 1800230.

62. Zhang, M., Qin, X., Gao, Y., Liang, J., Xiao, D., Zhang, X., Zhou, M., and Lin, Y. (2023). Transcutaneous Immunotherapy for RNAi: A Cascade-Responsive Decomposable Nanocomplex Based on Polyphenol-Mediated Framework Nucleic Acid in Psoriasis. Adv. Sci. *10*, e2303706.

63. Cai, Z., Li, Y., Bai, L., Xu, J., Liu, Z., Zhang, T., Gao, S., and Lin, Y. (2023). Tetrahedral Framework Nucleic Acids Based Small Interfering RNA Targeting Receptor for Advanced Glycation End Products for Diabetic Complications Treatment. ACS nano *17*, 22668–22683.

64. Zhang, X., Zhang, M., Zhou, M., Zhang, T., Gao, Y., Li, S., Lin, Y., and Cai, X. (2022). Tetrahedral-Framework Nucleic Acids Carry Small Interfering RNA to Downregulate Toll-Like Receptor 2 Gene Expression for the Treatment of Sepsis. ACS Appl. Mater. Inter. *14*, 6442–6452.

65. Gao, Y., Chen, X., Tian, T., Zhang, T., Gao, S., Zhang, X., Yao, Y., Lin, Y., and Cai, X. (2022). A Lysosome-Activated Tetrahedral Nanobox for Encapsulated siRNA Delivery. Adv. Mater. *34*, e2201731.

66. Zhang, T., Tian, T., Zhou, R., Li, S., Ma, W., Zhang, Y., Liu, N., Shi, S., Li, Q., Xie, X., et al. (2020). Design, fabrication and applications of tetrahedral DNA nanostructure-based multifunctional complexes in drug delivery and biomedical treatment. Nat. Protoc. *15*, 2728–2757.

67. Weill, D., Benden, C., Corris, P.A., Dark, J.H., Davis, R.D., Keshavjee, S., Lederer, D.J., Mulligan, M.J., Patterson, G.A., Singer, L.G., et al. (2015). A consensus document for the selection of lung transplant candidates: 2014–an update from the Pulmonary Transplantation Council of the International Society for Heart and Lung Transplantation. J. Heart Lung Transpl. *34*, 1–15.

68. Adams, T.S., Schupp, J.C., Poli, S., Ayaub, E.A., Neumark, N., Ahangari, F., Chu, S.G., Raby, B.A., DeIuliis, G., Januszyk, M., et al. (2020). Single-cell RNA-seq reveals ectopic and aberrant lung-resident cell populations in idiopathic pulmonary fibrosis. Sci. Adv. *6*, eaba1983.

69. Reyfman, P.A., Walter, J.M., Joshi, N., Anekalla, K.R., McQuattie-Pimentel, A.C., Chiu, S., Fernandez, R., Akbarpour, M., Chen, C.I., Ren, Z., et al. (2019). Single-Cell Transcriptomic Analysis of Human Lung Provides Insights into the Pathobiology of Pulmonary Fibrosis. Am. J. Respir. Crit. Care Med. *199*, 1517–1536.

YMTHE, Volume *32*

Supplemental Information

Tetrahedral DNA loaded siCCR2 restrains M1 macrophage polarization to ameliorate pulmonary fibrosis in chemoradiation-induced murine model

Chen Li, Xiaorong Feng, Songhang Li, Xing He, Zeli Luo, Xia Cheng, Jie Yao, Jie Xiao, Xiaofei Wang, Dingke Wen, Duanya Liu, Yanfei Li, Hong Zhou, Lu Ma, Tongyu Lin, Xiaoxiao Cai, Yunfeng Lin, Lu Guo, and Mu Yang

Supplemental Material

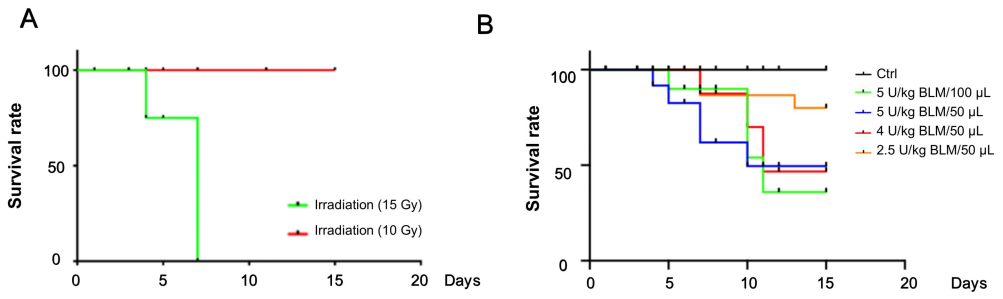

Figure S1. Does selection of IR and BLM. (A and B) Survival analyses after IR (A) and BLM (B) treatment in different dose.

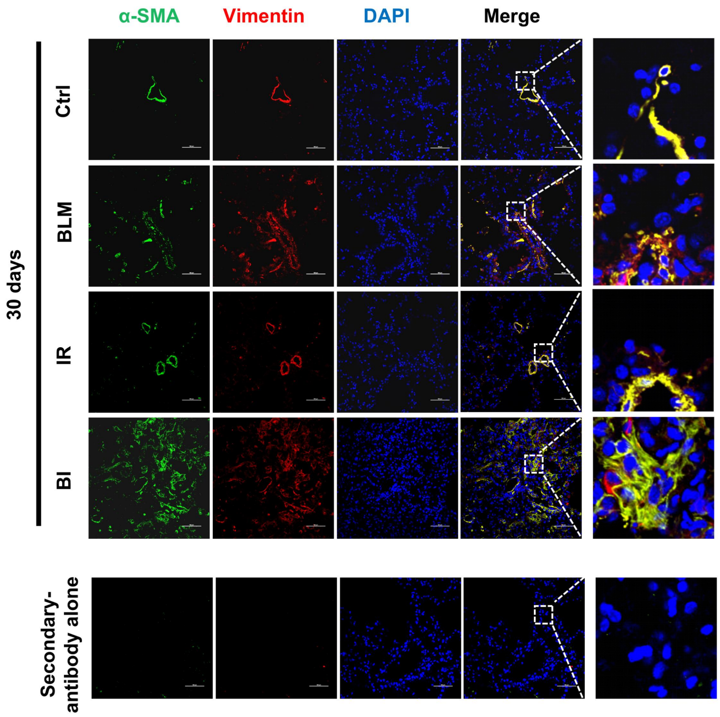

Figure S2. Activated myofibroblasts in pulmonary tissues on day 30 post-induction. Representative immunofluorescence images of α-SMA^{+}(green)Vimentin^{+}(red) myofibroblasts in pulmonary parenchyma of murine models; sections were co-stained by Vimentin (red) and α-SMA (green) antibodies, DAPI as blue nuclear counterstain, bar = 100 μm.

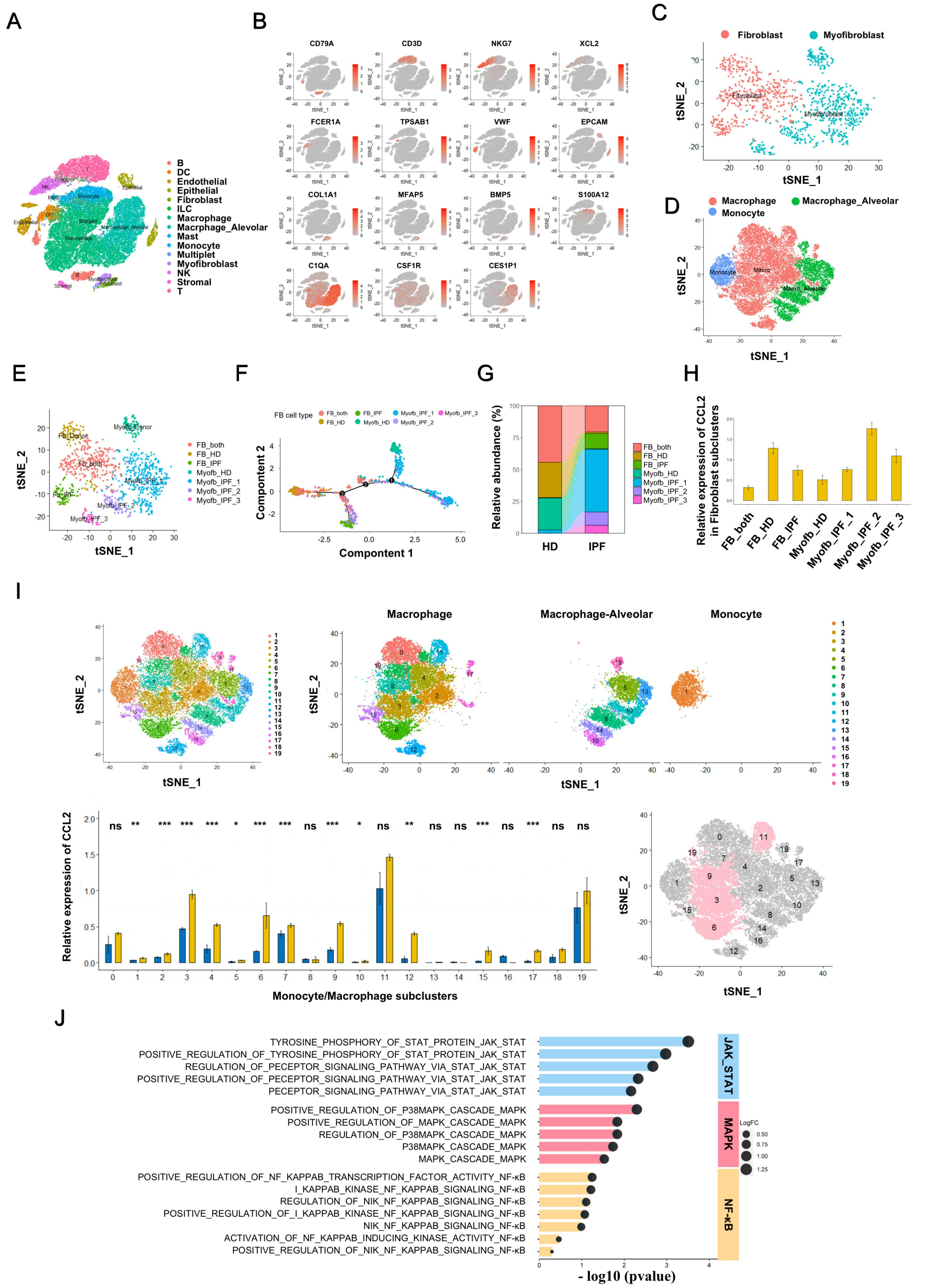

Figure S3. scRNAseq analysis of cell clusters in pulmonary parenchyma of IPF patients. (A) T-distributed stochastic neighbour embedding (t-STE) plot to visualize cell-type clusters based on unsupervised clustering analysis of scRNAseq. (B) Expression profile of selected genes in cell clusters of pulmonary parenchyma. (C)

Clusters of myofibroblast and fibroblast by individual annotation from scRNAseq data. (D) Clusters of monocytes and macrophages by individual annotation from scRNAseq data. (E) Sub-clusters of fibrosis/myofibrosis by individual annotation from scRNAseq data. (F) Pseudotime trajectory of fibrosis/myofibrosis sub-clusters analyzed by Monocle. (G) Relative abundance of fibrosis/myofibrosis sub-clusters. (H) CCL2 expression in selected sub-clusters of fibrosis/myofibrosis. (I) Sub-clusters of monocyte/macrophage by individual annotation from scRNAseq data, and CCL2 expression in selected sub-clusters. (J) KEGG analysis of CCL2/CCR2 axis in macrophage. Data were analyzed using student's *t*-test, $^{*}P < 0.05$, $^{**}P < 0.01$, and $^{***}P < 0.001$ versus positive control.

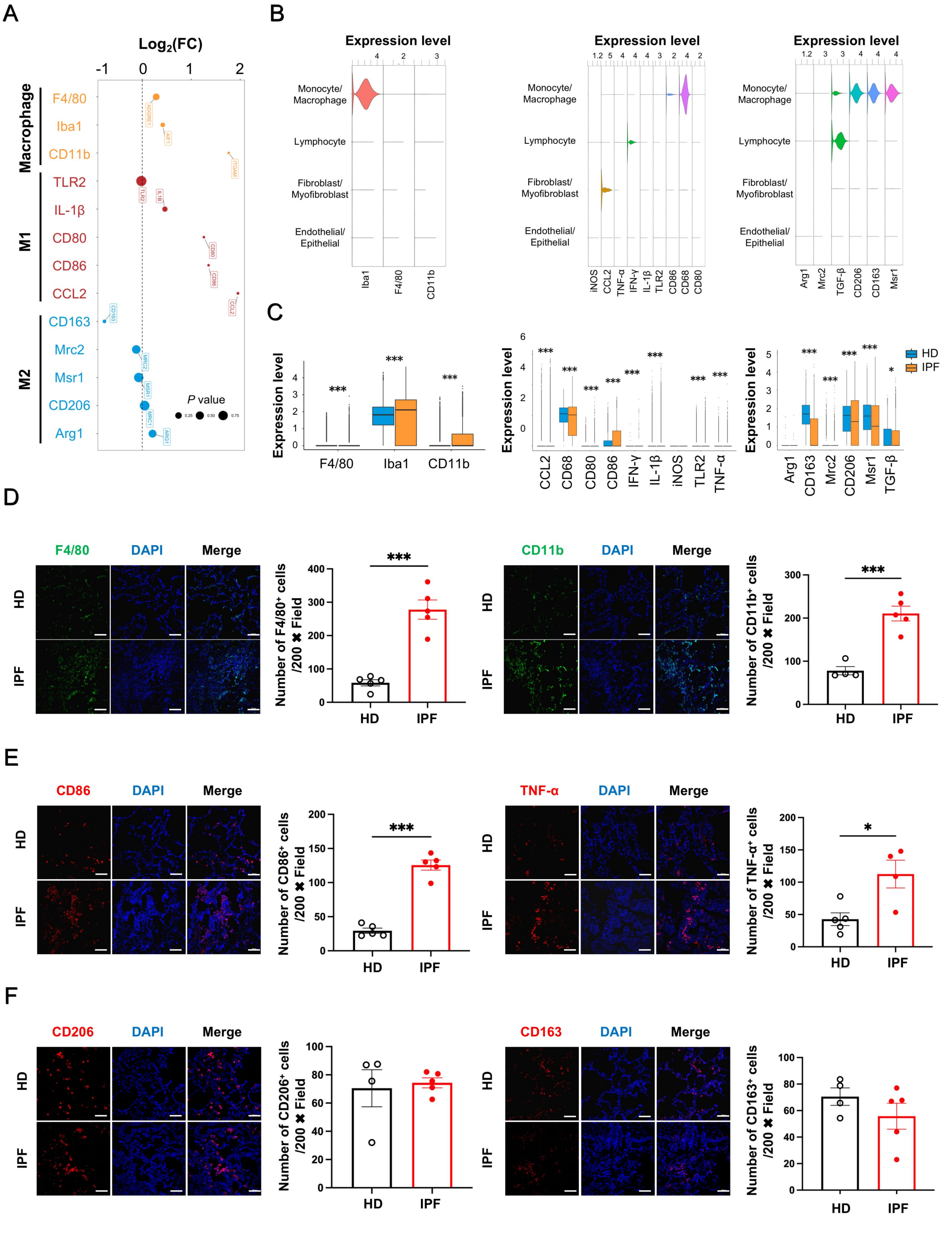

Figure S4. Identification of macrophage in pulmonary parenchymal of IPF patients. (A) Pesudo-bulk differential expression analysis of multiple macrophage parameters in lung between healthy donors and IPF patients (log_2(fold change(FC)) (GSE122960). (B) Multiple macrophage parameter expression levels in different cell clusters of lung. (C) Differential expression of multiple macrophage parameter in the monocyte/macrophage cluster of lung between healthy donors and IPF patients. (D)

Representative immunofluorescence images and quantification analysis F4/80$^+$ and CD11b$^+$ macrophage in lung of healthy donors and IPF patients. (E and F) Representative immunofluorescence images and quantification analysis of CD86$^+$ and TNFα$^+$ M1 macrophage (E) and CD206$^+$ and CD163$^+$(red) M2 macrophage (F) in lung sections of IPF patients and healthy donors. Bar=100 μm, data (n=4-5) were expressed as mean±SEM and analyzed using student's t-test, $^{*}P < 0.05$ and $^{***}P < 0.001$ between two groups.

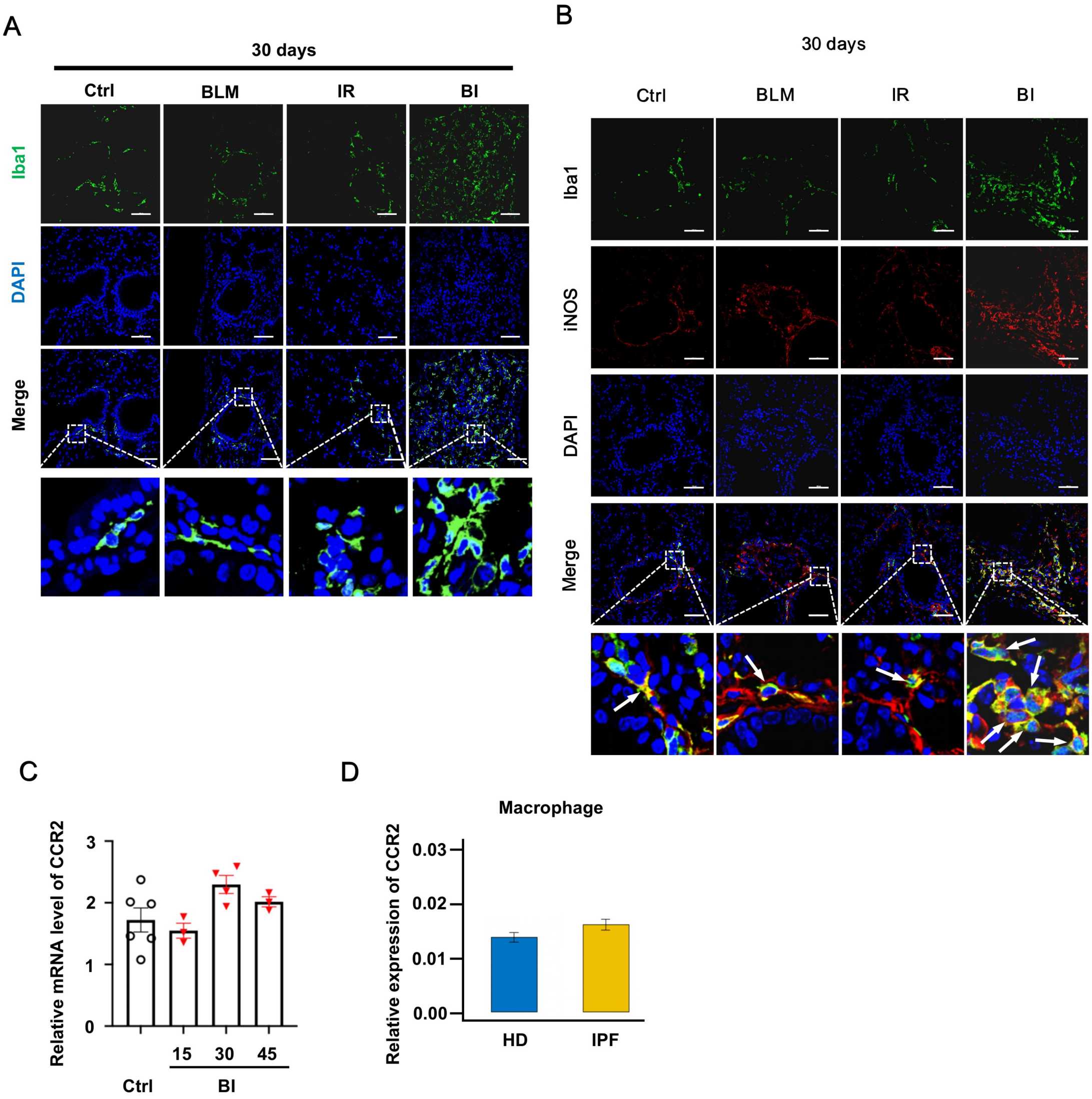

Figure S5. Increased M1 macrophage in pulmonary tissue of murine models on day 30 after induction. (A and B) Representative immunofluorescence images of Iba1^{+}(green) labeled macrophage (A) and Iba1^{+}(green)iNOS^{+}(red) labeled M1 macrophage (B) in pulmonary tissues. (C) The mRNA expression levels of CCR2 in pulmonary tissues from BI group at day 15, 30, and 45 post-induction. (D) The mRNA expression levels of CCR2 in macrophage of pulmonary tissues from HD and IPF patients. Bar=100 μm, data were expressed as mean±SEM and analyzed using one-way ANOVA with Tukey's multiple comparisons test.

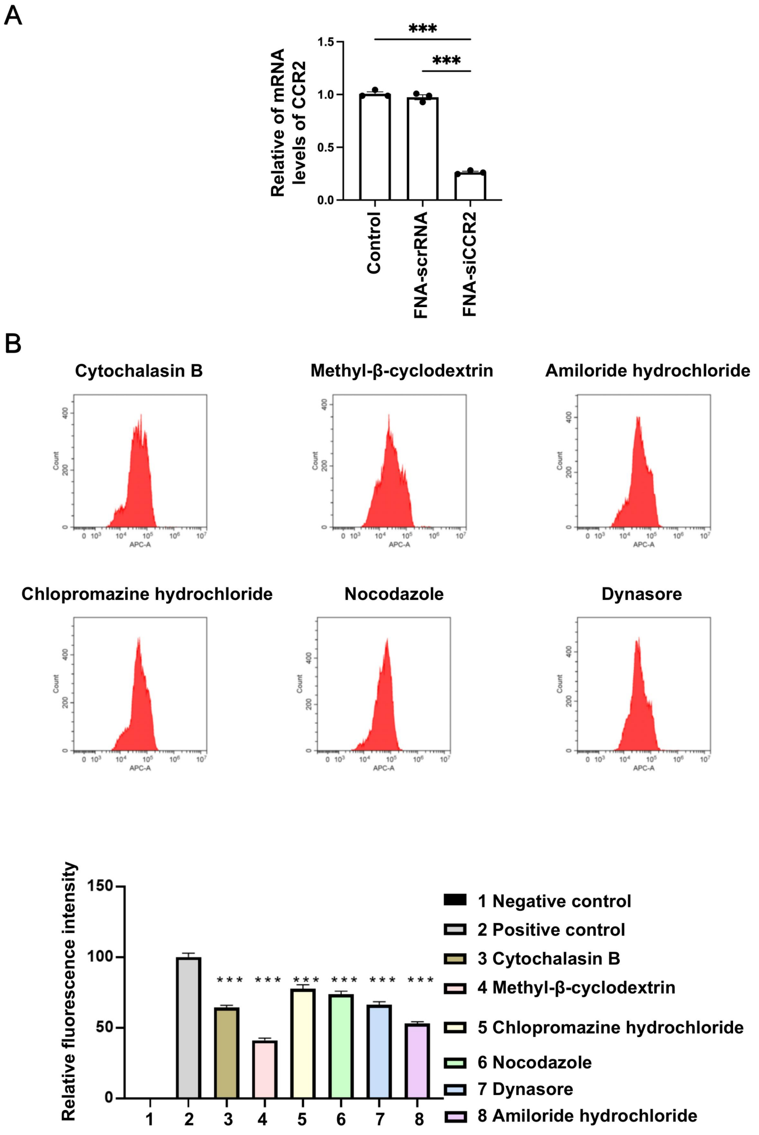

Figure S6. (A) RT-PCR reveal the inhibitory efficacy of FNA-siCCR2 on the CCR2 gene expression in macrophages. (B) Analysis of the amount of FNA-siCCR2-Cy5 endocytosed after adding various inhibitors, n=3 for each group. Data were expressed as mean±SEM and analyzed using student's t-test, $^{***}P < 0.001$ between two groups or versus positive control.

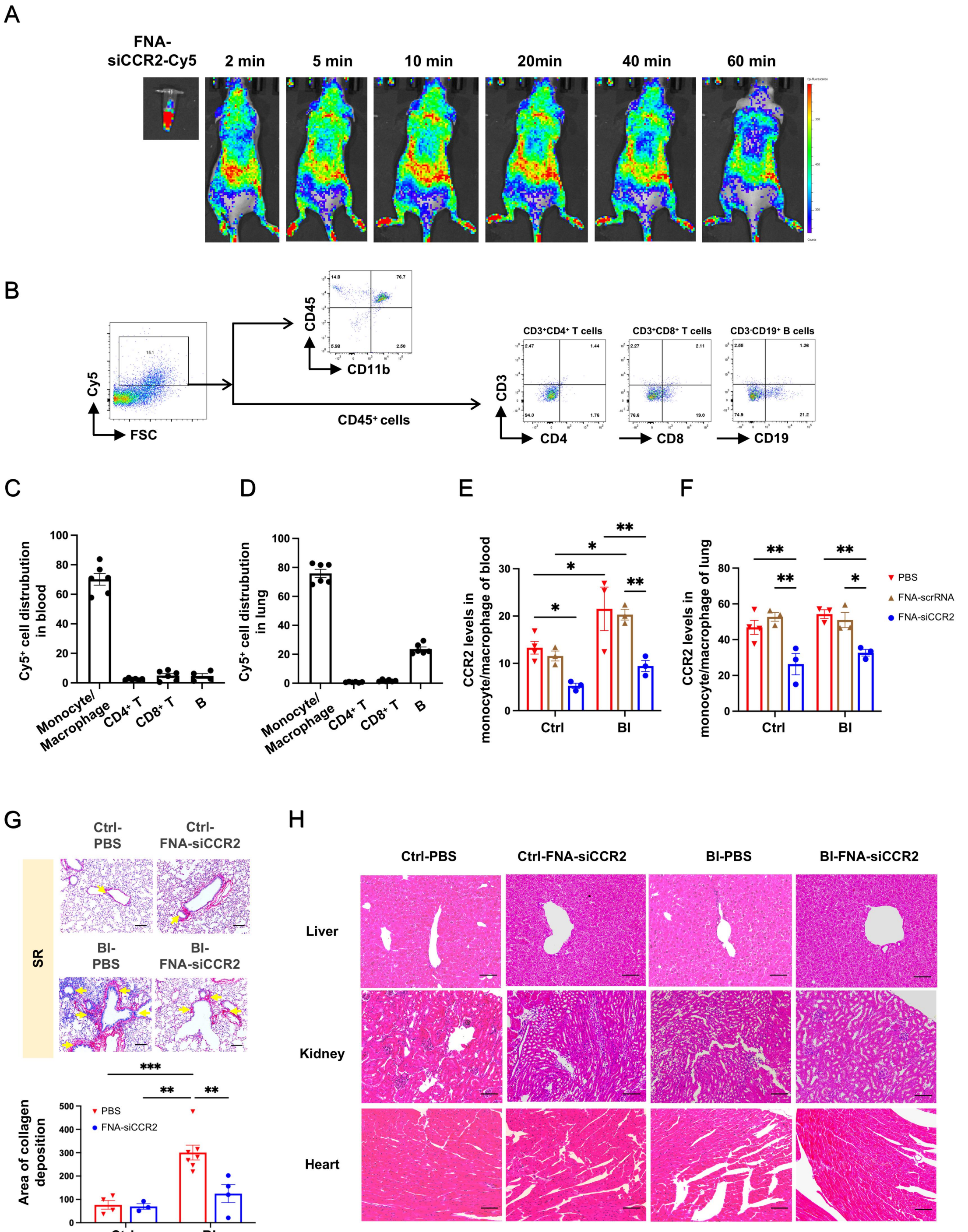

Figure S7. FNA-siCCR2 prevent pulmonary fibrosis in IPF murine model. (A) Continuously observation of FNA-siCCR2-Cy5 signals in mice after i.v. injection by in *vivo* imaging system. (B) Representative flow cytometry plots showing gating strategy for $CD45^+CD11b^+$ monocyte/macrophage, $CD3^+CD4^+$ T cells, $CD3^+CD8^+$ T cells, and $CD3^-CD19^+$ B cells in the PBMCs. (C and D) The cell distribution of $Cy5^+$ signals in blood and lung tissues after i.v. injection of FNA-siCCR2-Cy5. (E and F) Flow cytometry reveal the inhibitory efficacy of FNA-siCCR2 on the CCR2 gene

expression in monocyte/macrophages of blood and lung tissues. (G) The representative images of SR staining and statistically analysis of collagen deposition in lung tissues from murine IPF models after FNA-siCCR2 treated, bar=200 μm, n=3-7 for each group. (H) The representative images of HE staining of liver, kidney, and heart tissues from murine IPF model after FNA-siCCR2 treatment, bar=200 μm. Data were expressed as mean±SEM and analyzed using two-way ANOVA with Tukey's multiple comparisons test, $^{*}P < 0.05$ and $^{**}P < 0.01$ between two groups.

Table S1 Clinical details of idiopathic pulmonary fibrosis patients.

Patient NO.	Diagnosis	Gender	Age (y)	Duration (y)	BMI	Smoker	Oxygenation index (mmHg)	6 min walk distance (m)
P1	IPF	Male	59	6	27.38	No	133.33	NA
P2	IPF	Male	57	0.8	16.53	Yes	165.85	120
P3	IPF	Female	65	2	23.73	No	184.44	135
P4	IPF	Male	69	2	24.91	Yes	165.85	NA
P5	IPF	Male	62	1.6	21.22	Yes	248.65	NA

NA: Not available

Table S2 Clinical details of healthy donors.

Healthy donor NO.	Gender	Age (y)	Oxygenation index (mmHg)	6 min walk distance (m)
HD1	Male	58	385.71	480
HD2	Male	72	360.71	423
HD3	Female	63	382.14	400
HD4	Male	64	375	468
HD5	Male	66	417.86	416

Table S3 Clinical details of donors for scRNAseq from GSE136831.

	Samples NO.	Diagnosis	Gender	Age
1	GSM4058905	Control	M	50
2	GSM4058907	Control	M	56
3	GSM4058911	Control	M	65
4	GSM4058912	Control	M	64
5	GSM4058914	Control	F	66
6	GSM4058915	Control	F	66
7	GSM4058916	Control	M	61
8	GSM4058917	Control	F	62
9	GSM4058918	Control	M	35
10	GSM4058921	Control	F	67
11	GSM4058923	Control	F	66
12	GSM4058926	Control	M	54
13	GSM4058948	IPF	M	56
14	GSM4058950	IPF	F	66
15	GSM4058952	IPF	F	67
16	GSM4058954	IPF	F	65
17	GSM4058957	IPF	F	67
18	GSM4058960	IPF	M	56
19	GSM4058961	IPF	M	61
20	GSM4058963	IPF	M	65
21	GSM4058969	IPF	M	64
22	GSM4058972	IPF	F	66
23	GSM4058974	IPF	M	54
